# Cochlear transcriptome analysis of an outbred mouse population (CFW)

**DOI:** 10.3389/fncel.2023.1256619

**Published:** 2023-11-29

**Authors:** Ely Cheikh Boussaty, Neil Tedeschi, Mark Novotny, Yuzuru Ninoyu, Eric Du, Clara Draf, Yun Zhang, Uri Manor, Richard H. Scheuermann, Rick Friedman

**Affiliations:** ^1^Department of Otolaryngology, University of California, San Diego, La Jolla, CA, United States; ^2^J. Craig Venter Institute, La Jolla, CA, United States; ^3^Department of Cell and Developmental Biology, University of California San Diego, Salk Institute for Biological Studies, Waitt Advanced Biophotonics Center, La Jolla, CA, United States; ^4^Department of Pathology, University of California, San Diego, La Jolla, CA, United States

**Keywords:** single nucleus RNA sequencing, cell ontology, marker genes, age-related hearing loss, genetic determinants of hearing loss

## Abstract

Age-related hearing loss (ARHL) is the most common cause of hearing loss and one of the most prevalent conditions affecting the elderly worldwide. Despite evidence from our lab and others about its polygenic nature, little is known about the specific genes, cell types, and pathways involved in ARHL, impeding the development of therapeutic interventions. In this manuscript, we describe, for the first time, the complete cell-type specific transcriptome of the aging mouse cochlea using snRNA-seq in an outbred mouse model in relation to auditory threshold variation. Cochlear cell types were identified using unsupervised clustering and annotated via a three-tiered approach—first by linking to expression of known marker genes, then using the NSForest algorithm to select minimum cluster-specific marker genes and reduce dimensional feature space for statistical comparison of our clusters with existing publicly-available data sets on the gEAR website,^1^ and finally, by validating and refining the annotations using Multiplexed Error Robust Fluorescence *In Situ* Hybridization (MERFISH) and the cluster-specific marker genes as probes. We report on 60 unique cell-types expanding the number of defined cochlear cell types by more than two times. Importantly, we show significant specific cell type increases and decreases associated with loss of hearing acuity implicating specific subsets of hair cell subtypes, ganglion cell subtypes, and cell subtypes within the stria vascularis in this model of ARHL. These results provide a view into the cellular and molecular mechanisms responsible for age-related hearing loss and pathways for therapeutic targeting.

## Introduction

Progressive, bilateral sensorineural hearing impairment affects approximately 25% of people aged 65–74 and 50% aged 75 and older. In the United States, two thirds of people over 70 years of age have some degree of hearing loss (Bainbridge and Wallhagen, 2014; Gurgel et al., 2014; Jayakody et al., 2018). Aside from the detrimental impact on quality of life, hearing loss also carries an increasing economic burden as the cost of medical expenditures is expected to reach $60 billion in 2030. It is projected that 2.45 billion people will have hearing loss by 2050, a 56.1% increase from 2019, despite stable age-standardized prevalence (Sheffield and Smith, 2019; Vos et al., 2020). Notably, a substantial fraction of patients with progressive hearing loss have no identifiable mutation in any known hearing loss gene among the 100+ that have been identified, suggesting that a significant fraction of hearing loss is due to unidentified monogenic or polygenic causes (Bowl and Dawson, 2019). While there is notable differences in developmental stages between mouse and humans, mainly due length of theirs lifespan, there is a correlation between hearing loss i.e., a 2-year-old mouse is equivalent to a ~ 70-year-old human and the corresponding age to which hair cells are lost, leading to a particularly profound hearing loss in a mouse which also depends upon the mouse strain (Elliott et al., 2022, for review).

As part of a broad approach to studying the genetic landscape of ARHL, we have begun a largescale effort to phenotypically characterize the auditory function of young and aged Carworth Farms White (CFW) Crl:CFW(SW)-US_P08 (hereafter CFW) outbred mice (Parker et al., 2016). Although not specifically developed for genetic research, these mice have several attractive properties for gene discovery. CFW mice were derived from a small number of founders and have been maintained as an outbred population for more than 100 generations, thus, reducing the size of linkage disequilibrium between alleles (Rice and O’Brien, 1980).

Developing therapies for progressive hearing loss necessitates an understanding of the genes and pathways involved. The cellular complexity of the inner ear, including the sensory and supporting cells of the organ of Corti, the lateral wall (stria vascularis), and the auditory neurons, necessitates a single cell approach to gain an understanding of the pathway changes associated with hearing loss. Due to this complexity, a few laboratories have used single-cell RNA sequencing (scRNAseq) to characterize the molecular mechanisms underlying cochlear development (de Bruijn and Dzierzak, 2017) and to gain insights into aging in a single mouse strain, and after acoustic trauma (Petitpré et al., 2018, 2022; Shrestha et al., 2018; Sun et al., 2018; Korrapati et al., 2019; Hoa et al., 2020; Kolla et al., 2020; and more recently Jean et al., 2023). This approach has provided insights into the molecular mechanisms underlying cochlear development (Brown et al., 2008) and response to damaging noise (Lavinsky et al., 2015; Liu et al., 2021). Recently, Li et al. (2018) and Liu et al. (2022) looked at the transcriptome of inner and outer hair cells and the stria vascularis in aging CBA/J mice, implicating changes in several processes including gene transcription, DNA damage, autophagy, and oxidative stress. However, these single cell analyses of the adult mouse inner ear, particularly the organ of Corti, have been hampered by the difficulty in dissociating cells from the tissue due to their tight junction connections and their extracellular matrix embeddings (Burns et al., 2015).

In this study, we used *single nucleus RNA-seq (snRNA-seq)* across 48 genetically diverse CFW outbred mice at 10 months of age to provide for a more unbiased representation of cell types associated with variations in hearing. We identified gene expression signatures for 60 distinct cell types withing the cochlea, including novel markers for inner and outer hair cell subtypes and found that specific hair cell, ganglion, and stria vascularis cell types show preferential depletion associated with hearing loss. To our knowledge this is the first snRNA-seq study to examine differential gene expression across all cochlear cell types in genetically diverse outbred mice with varying degrees of hearing loss.

## Materials and methods

### Animals

Animals originated from the same stock of outbred mice Crl:CFW(SW)-US_P08 (CFW), maintained by Charles River Laboratories (CRL) in Portage, Michigan, were used in this study. All procedures were performed in accordance with guidelines from NIH and with approval by the Institutional Care and Use Committee (IACUC) at University of California San Diego (IACUC S17178).

### Auditory phenotyping/hearing patterns determination

All CFW mice were subsequently aged until a final age of 10 months. Within this period, phenotypic audiometric assessment using Auditory Brainstem Response (ABR) was completed at three time points: young adults (5–8 weeks), 6 months, and 10 months. Auditory phenotyping and determination of hearing patterns were previously detailed in Du et al. (2022). Briefly, upon aging to the stated time points, anesthetized mice were presented to auditory signals as tone pips ranging from 20 to 100 dB SPL at the frequencies 4, 8, 12, 16, 24, and 32 kHz. The detection distinctive ABR waveform at each frequency was used to determine the hearing thresholds and characterize ARHL within the CFW mice into one of eight distinct hearing patterns: Normal hearing; isolated mid-frequency (12 or 16 kHz) loss; Moderate high-frequency (24 or 32 kHz) loss; Severe high-frequency (only 32 kHz) loss; Severe high-frequency (24 + 32 kHz) loss; Moderate all-frequency loss; Severe all-frequency loss; and Profound all-frequency loss.

### Isolation of cochlear tissue

A group of 48 mice were sacrificed and their inner ears were utilized to generate single nucleus RNA-seq transcriptomes. Mice were selected to represent the hearing pattern group described above based on the proportion of such pattern in the general CFW cohort. Special care was taken to complete the microdissection of the two cochleae from each mouse in less than 7 min while ensuring a minimum physical stretching on the organ of Corti. Samples were collected at the same time of day across individual and batches.

Following the ABR testing at 10 months age final time point, the anesthetized mice were decapitated, and their inner ears were quickly transferred into ice-cold DPBS buffer (Thermo Fisher Scientific) for microdissection. Tissue from utricule and saccule were carefully removed to avoid including unwanted vestibular cell types before removing the bony wall of the cochlea by breaking chopping out the surrounding bone in pieces and freeing the cochlear tissue. The microdissected tissue from each mouse was pooled in a tube containing 1 mL of Iscove’s Modified Dulbecco’s Medium (IMDM; Thermo Fisher Scientific).

### Single nucleus isolation and RNA sequencing

Immediately after isolation, bulk cochlear tissues in cell culture media were processed for snRNAseq serially in sets of four specimens and kept on ice for the entire procedure. Dounce homogenization was performed to isolate individual nuclei from the bulk cochlear tissue followed by Fluorescent Automated Cell Sorting (FACS) to provide purified, intact nuclei for RNA-seq as detailed in Krishnaswami et al. (2016) with the following modifications. Briefly, a total of 5 mL of Homogenization Buffer with Propidium Iodide (PI; 1.5 μM, Thermo Fisher cat. #P3566) and Calcien, AM (1.0 μM; Thermo Fisher cat. #C1430) was used to stain intact nuclei and exclude whole cells from being sorted. The cochlear tissue in cell culture media was briefly centrifuged to remove the supernatant to approximately 100 μL volume and the remaining tissue transferred using a 1 mL wide bore pipette tip into 1 mL of chilled Homogenization Buffer in a Dounce Homogenizer (Wheaton cat. #357538) stored on ice. A total of 10 strokes with the loose piston followed by 14 strokes with the tight piston was followed by filtration through a series of two cell strainers (Becton Dickenson Falcon cat. #352235) and loading onto a Beckton Dickenson FACS Aria II cell sorter with a 70 mm nozzle. A chilled 96-well plate was used with 4 μL of Homogenization Buffer placed in wells A1–A4 as a destination for sorted nuclei. FACS gating of nuclei were accomplished with forward and side scatter doublet nuclei gating exclusion, green channel (Calcien, AM) exclusion, and triggered on the red (PI) channel with approximately 1–7% of the total event population consisting of intact nuclei. A total of 36,000 sorted nuclei from each of the four samples were sorted into wells A1–A4 with a final volume of approximately 60 μL after completion of sorting.

Isolated nuclei in the four wells were kept on ice and processed for RNA-seq using the Chromium Next GEM Chip G Single Cell Kit (10X Genomics cat. #1000120) according to manufactures instructions with the following modifications. A total of 43.2 μL of sorted volume containing approximately 26,000 nuclei was added to 31.8 μL of the Single Cell Master mix for a total of 75 μL, followed by loading of 70 μL (approx. 24,267 nuclei) onto the Next GEM chip. cDNAs were amplified for 13 cycles. Recovered cDNAs were quality controlled by loading 1 μL of cDNA onto an Agilent Bioanalyzer using a High Sensitivity DNA chip kit (cat. #5067-4626), followed by library prep according to manufacturer’s instructions for dual index barcoded libraries.

The nuclei isolation, sorting, and Next Gem chip cDNA synthesis procedures were repeated 12 more times for a total of 48 cDNA library samples. A total of 48 single nuclei barcoded RNA-seq libraries were subsequently generated from the cDNAs using the Single Index Kit T Set A (10X Genomics cat. #1000213) and 16 cycles of library amplification followed by purification according to manufactures instructions. A 1 μL loading of a 10-fold dilution of each library was performed for quality control and quantification on an Agilent High Sensitivity chip. Equimolar amounts (6 nM) of a subset of 16 libraries were pooled and diluted to create a final loading of a 300 pM library pool onto a dual-lane NovaSeq 6000 (Illumina, Inc.) using the S2 Reagent Kit v1.5 Paired End 2 × 50 base (100 cycle) sequencing kit (Illumina cat. #20028316). Sequencing parameters were set for R1 at 28 cycles, I7 Index at 8 cycles, I5 index at 0 cycles, and Read 2 at 91 cycles for a calculated R2 read count of 25,600 per cell. Two more NovaSeq 6000 S2 runs of 16 library pools were subsequently loaded for a total of three runs to complete the RNA-seq of all 48 samples. A range of X to Y reads per cell were generated.

### Read alignment

The cellranger count command with the—include-intron option from the 10x Cell Ranger 5.0.1 package (Zheng et al., 2017) was used to align reads and count barcodes and UMIs. Reads were aligned to the Cell Ranger reference package refdata-gex-mm10-2020-A.

### Quality control

Data quality was assessed using the number of nuclei per sample, the number of UMIs (library size), the number of genes detected, and the number of mitochondrial genes per nuclei. Nuclei were removed if they had fewer than 1,000 UMIs and/or fewer than 500 detected genes. Nuclei that had the number of detected mitochondrial genes tagged as outliers by the isOutlier function from the scuttle package were also removed (McCarthy et al., 2017).

### Normalization, feature selection, and dimensionality reduction

UMI counts were normalized using logNormCounts from the scuttle package. Highly variable genes were selected using modelGeneVar and getTopHVGs functions from the scran package (Lun et al., 2016). PCA was performed using the runPCA function from the scater package. Nineteen principal components were used for clustering, which accounted for 50% of the variance.

### Clustering and doublet detection

To minimize the inclusion of droplets that have more than one nucleus, putative doublets were identified using the scDblFinder package (Germain et al., 2021). Since scDblFinder uses cluster information to predict doublets, nuclei were first clustered using the Leiden community detection algorithm as implemented in the leidenalg python package (Traag et al., 2018). First, a shared nearest neighbor graph was constructed with buildSNNGraph from the scran package (McCarthy et al., 2017). Then, the leidenalg.find_partition function with partition_type set to ModularityVertexPartition was called. Finally, sclDblFinder was called using the resultant cluster labels. About 6% of the droplets were predicted to be doublets and removed from downstream analysis (Supplementary Figure 6). The remaining nuclei were re-processes repeating quality control, normalization, feature selection, dimensionality reduction, and clustering.

### Visualization

The clustered data set was visualized by running runUMAP and runTSNE from the scater package (McCarthy et al., 2017). For runUMAP, n_neighbors was set to 100; for runTSNE, perplexity was set to 100.

### Sub-clustering

**Figure 1:**
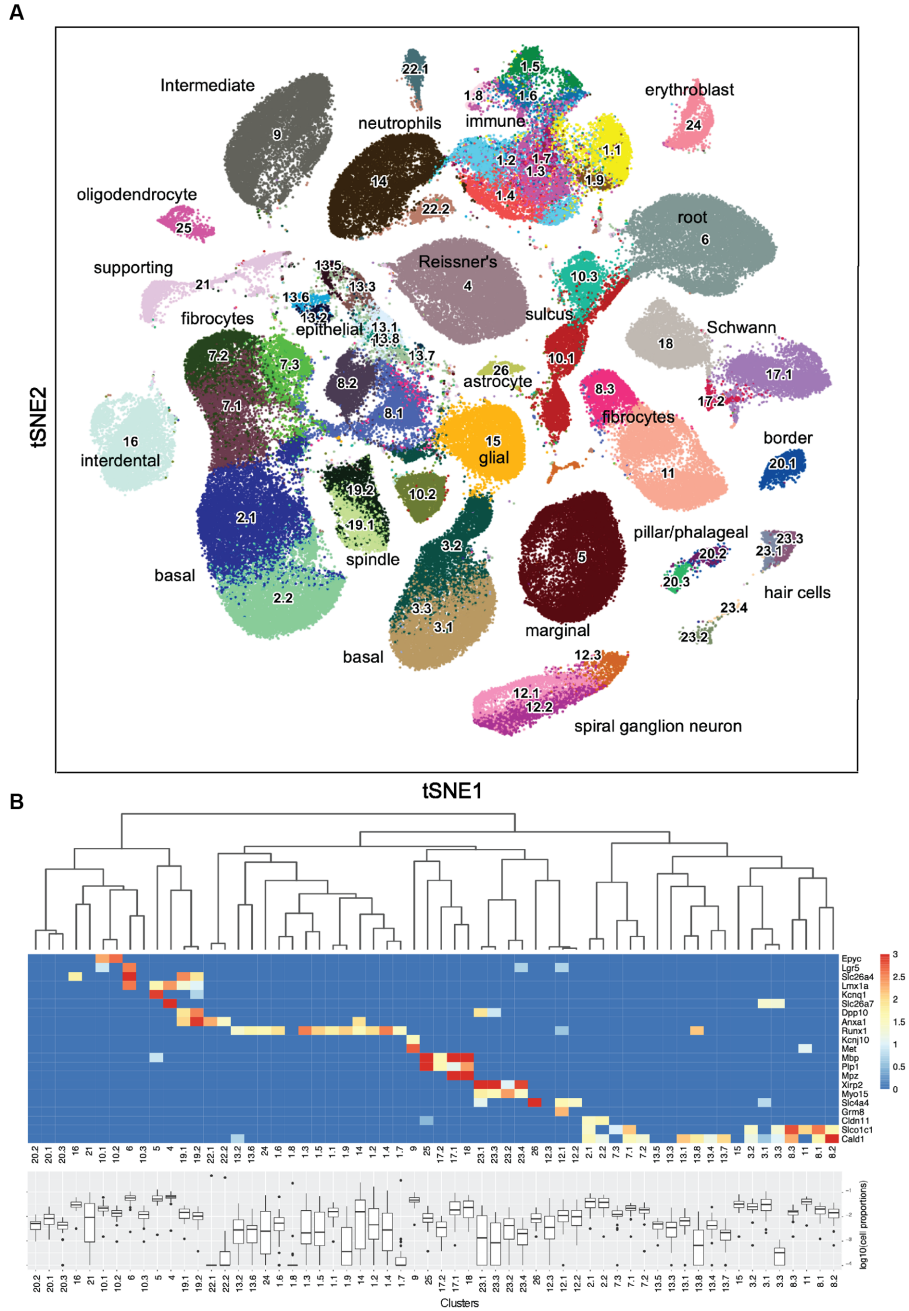
Sixty snRNA-seq transcriptomic clusters from mouse cochlea. **(A)** Iterative unsupervised clustering of the processed cell-by-gene snRNA-seq expression matrix performed using Leiden community detection identified 60 distinct transcriptomic clusters (numbers). tSNE embedding of the clustering results is shown. Cell type annotations of each transcriptomic cluster based on subsequent analysis (Table 1) are shown. The data can be explored at https://umgear.org/index.html?multigene_plots=0&layout_id=c2acc279&gene_symbol_exact_match=1&gene_symbol=slc26a5. **(B)** Heatmap of marker genes known to be expressed in specific ear cell types arranged according to hierarchical clustering of transcriptomic clusters, which captures the transcriptional relationships between the discrete clusters. The horizontal banding patterns of these known marker genes validate the structure of the hierarchical clustering taxonomy. Box plots of the proportion (log10 transformed) of each cell type across the 48 outbred mouse cochlear samples, with median proportion ranging from ~10−4 for Cluster 22.1 to ~10−1 for Cluster 4.

The initial clustering using Leiden community detection yielded 26 clusters. To determine if any of these clusters should undergo another round of clustering, two methods were used: the silhouette width, which compares the average distance of a nucleus to all other nuclei within the same cluster to the average distance to nuclei in the nearest neighboring cluster (Amezquita et al., 2021; Section 5.2.2), and manual inspection. Supplementary Figure 7 shows an example of this analysis where Clusters 1, 10, and 17 contain separate areas of nuclei with negative silhouette widths colored in red as candidates for sub-clustering. Other candidate clusters were detected by manual inspection of the tSNE embeddings (Figure 1A), for example, Clusters 20 and 23, which appear to have two distinct “islands” suggesting the need for sub-clustering. Supplementary Figure 8 shows examples of the resulting subclusters. By applying those criteria on the initial clusters, 13 were retained and 13 went through a second round of sub-clustering, which brought the total number of transcriptomic clusters identified to 60. The final clustered cell-by-gene expression matrix can be found at the gEAR resource—https://umgear.org/p?l=c2acc279.

**Table 1:** Analysis and annotations of transcriptomics clusters using FR-Match. Comparison of transcriptomics clusters against five recently released snRNA and scRNA postnatal using the cluster-specific marker genes identified by NS-Forest.

Hierarchical cluster order	Cluster ID	Cluster name	Cell type name	FR-Match high confidence	FR-Match low confidence	MERFISH cell type|anatomic structure	enrichR
38	**26**	Cochlear astrocyte	Cochlear astrocyte Gabrb1 Slc39a12		Hensens	Astrocyte| Cochlear nerve	Astrocyte
22	**1.5**	Cochlear B cell	Cochlear B cell Chst3 Pax5	B cell	B cell	B cell | Cochlear nerve	B cell
42	**2.1**	Cochlear basal 1	Cochlear basal Sorcs3 Spaar	Basal	Basal	Basal cell | Spiral ligament	
43	**2.2**	Cochlear basal 2	Cochlear basal Dync1i1 Gm12153	Basal	Basal	Basal cell | Cochlear ganglion	
54	**3.2**	Cochlear basal 3	Cochlear basal Bnc2 Fn1	Fibrocyte	Basal	Basal cell | Cochlea	
55	**3.1**	Cochlear basal 4	Cochlear basal Slc47a1 Slc4a10	Fibrocyte	Basal	Basal cell | Cochlea	
56	**3.3**	Cochlear basal 5	Cochlear basal Clic4 Gm26740	Fibrocyte	Basal	Basal cell | Cochlea	
2	**20.1**	Cochlear border cell	Cochlear border cell Dgkb Klhl14		root	Border cell of cochlea | Epithelium of cochlear duct	Mixed
20	**1.8**	Cochlear CD8+ T cells	Cochlear CD8+ T cells Runx3 Grap2			CD8-positive, alpha-beta T cell | cochlea	CD8+ T cells
48	**13.3**	Cochlear ciliated epithelial 1	Cochlear ciliated epithelial Lgr6 Fam78b	Fibrocyte	Fibrocyte	Ciliated epithelial cell |??	Ciliated epithelial/osteocytes
49	**13.1**	Cochlear ciliated epithelial 2	Cochlear ciliated epithelial Satb2 Fap	Fibrocyte	Fibrocyte	Ciliated epithelial cell | Cochlear modiolus	Ciliated epithelial
50	**13.8**	Cochlear ciliated epithelial 3	Cochlear ciliated epithelial Cxcl12 Svep1	Fibrocyte	Fibrocyte	Ciliated epithelial cell | Cochlear modiolus	Ciliated epithelial/fibroblast
51	**13.4**	Cochlear ciliated epithelial 4	Cochlear ciliated epithelial Mecom	Fibrocyte	Fibrocyte	Ciliated epithelial cell |??	Ciliated epithelial
47	**13.5**	Cochlear endothelial	Cochlear endothelial Ptprb	Fibrocyte	Fibrocyte	Endothelial cell | Spiral modiolar artery	Ciliated epithelial/endothelial
18	**24**	Cochlear erythroblast	Cochlear erythroblast Kel	Erythroblast	Erythroblast	Erythroblast | Cochlea	Erythroblast
44	**7.3**	Cochlear fibrocyte 1	Cochlear fibrocyte Kcnk2	Fibrocyte	Fibrocyte	Fibrocyte | Spiral ligament	
45	**7.1**	Cochlear fibrocyte 2	Cochlear fibrocyte Slc8a3 Ucma	Fibrocyte	Fibrocyte	Fibrocyte | Spiral ligament	
46	**7.2**	Cochlear fibrocyte 3	Cochlear fibrocyte Slc4a10 Slc4a11	Fibrocyte	Fibrocyte	Fibrocyte | Spiral ligament	
57	**8.3**	Cochlear fibrocyte 4	Cochlear fibrocyte Themis Itga8	Fibrocyte	Fibrocyte	Fibrocyte | Cochlear ganglion	
58	**11**	Cochlear fibrocyte 5	Cochlear fibrocyte Mybpc1 Nav3	Fibrocyte	Fibrocyte	Fibrocyte | Basilar membrane of cochlea	Various stromal cell types
59	**8.1**	Cochlear fibrocyte 6	Cochlear fibrocyte Sorcs3 Lama2	Fibrocyte	Fibrocyte	Fibrocyte | Cochlear ganglion	
60	**8.2**	Cochlear fibrocyte 7	Cochlear fibrocyte Itga8 Slit2	Fibrocyte	Fibrocyte	Fibrocyte | Spiral ligament/cochlear ganglion	
53	**15**	Cochlear glial	Cochlear glial Slc6a13	Fibrocyte	Fibrocyte	Glial cell | Cochlear nerve	Glial
35	**23.3**	Cochlear hair cell 1	Cochlear hair cell Ush2a C230072F16Rik			Cochlea auditory hair cell | Epithelium of cochlear duct	Neuron/ganglion
37	**23.4**	Cochlear hair cell 2	Cochlear hair cell Ripor3			Cochlea auditory hair cell | Epithelium of cochlear duct	Mixed
34	**23.1**	Cochlear inner hair cell	Cochlear inner hair cell Gm1113 Ofcc1			Cochlea inner hair cell | Epithelium of cochlear duct	Myocytes
4	**16**	Cochlear interdental cell	Cochlear interdental cell Otoa Ceacam16		Root/Deiter	Interdental cell of cochlea | Tympanic lip of limbus of osseous spiral lamina	Lymphoid
29	**9**	Cochlear Intermediate	Cochlear Intermediate Dct	Intermediate	Intermediate	Strial intermediate cell | Stria vascularis of cochlear duct	Melanocytes
21	**1.3**	Cochlear leukocyte 1	Cochlear leukocyte Rnf220 Bcl11a			Leukocyte | Cochlea	
27	**1.4**	Cochlear leukocyte 2	Cochlear leukocyte Rab44 Cdk6			Leukocyte | Cochlea	
23	**1.1**	Cochlear macrophage 1	Cochlear macrophage 1	Macrophage	Macrophage	Tissue-resident macrophage | Cochlea	Various immune cell types
24	**1.9**	Cochlear macrophage 2	Cochlear macrophage 2 Spic			Tissue-resident macrophage | Cochlea	Macrophage
10	**5**	Cochlear marginal	Cochlear marginal Stac Kcnq1	Marginal	Marginal	Strial marginal cell | Stria vascularis of cochlear duct	Epithelial
28	**1.7**	Cochlear myeloid/neutrophil	Cochlear myeloid/neutrophil Gm20528 Tspoap1			Myeloid leukocyte | Cochlea	Myeloid/neutrophil
14	**22.1**	Cochlear neutrophil 1	Cochlear neutrophil S100a8 Hba-a1		Neutrophil	Neutrophil | Cochlea	Neutrophils
15	**22.2**	Cochlear neutrophil 2	Cochlear neutrophil S100a8 Retnlg	Neutrophil	Neutrophil	Neutrophil | Cochlea	
25	**14**	Cochlear neutrophil 3	Cochlear neutrophil Abca13 Adpgk	Neutrophil	Neutrophil	Neutrophil | Cochlea	Myeloid/neutrophil
26	**1.2**	Cochlear neutrophil 4	Cochlear neutrophil F13a1 Lyn			Neutrophil | Cochlea	
19	**1.6**	Cochlear NK/T cell	Cochlear NK/T cell Gm2682 Skap1			Mature NK T cell | Cochlear nerve	NK/T cell
30	**25**	Cochlear oligodendrocyte	Cochlear oligodendrocyte Prr5l Mog		Oligodendrocyte	Oligodendrocyte | Cochlear nerve	Oligodendrocyte
36	**23.2**	Cochlear outer hair cell	Cochlear outer hair cell Slc26a5	Outer hair cell	Outer hair cell	Cochlear outer hair cell | Epithelium of cochlear duct	Mixed
3	**20.3**	Cochlear phalageal cell	Cochlear phalageal cell Dgkb Nckap5		Root	Inner phalangeal cell/Deiters’ cell | Epithelium of cochlear duct	
1	**20.2**	Cochlear pillar cell	Cochlear pillar cell Mdm1		Root	Inner/outer pillar cell | Epithelium of cochlear duct	Mixed
11	**4**	Cochlear Reissner’s membrane	Cochlear Reissner’s membrane Gm48447	Reissner’s	Reissner’s	Epithelial cell | Membrane of Reissner	Retinal/sensory
8	**6**	Cochlear root cell	Cochlear root cell Lgr5 Slc26a4	Root	Root	Root cell | Outer spiral sulcus	
31	**17.2**	Cochlear Schwann cell 1	Cochlear Schwann cell Ntng1 Cdh19			Schwann cell | Cochlear ganglion/cochlear nerve	Stromal
32	**17.1**	Cochlear Schwann cell 2	Cochlear Schwann cell Pde1c Lama1			Schwann cell | Cochlear ganglion/cochlear nerve	Neuron
33	**18**	Cochlear Schwann cell 3	Cochlear Schwann cell Mpz Gm12068	Glia	Glia/Schwann cell	Schwann cell | Cochlear ganglion/cochlear nerve	Schwan cell
52	**13.7**	Cochlear smooth muscle cell	Cochlear smooth muscle cell Mrvi1 Trpc3	Fibrocyte	Fibrocyte	Smooth muscle cell | Spiral modiolar artery	Ciliated epithelial/smooth muscle
12	**19.1**	Cochlear spindle 1	Cochlear spindle Gm43154 Agbl1	Spindle	Spindle	Spindle cell | Spiral prominence of cochlear duct	
13	**19.2**	Cochlear spindle 2	Cochlear spindle Dpp10 Anxa1	Spindle	Spindle	Spindle cell | Spiral prominence of cochlear duct	
39	**12.3**	Cochlear spiral ganglion neuron 1	Cochlear spiral ganglion neuron 3 Meg3			Spiral ganglion neuron | Cochlear ganglion	
40	**12.1**	Cochlear spiral ganglion neuron 2	Cochlear spiral ganglion neuron 1 C130073E24Rik Ntng1			Spiral ganglion neuron | Cochlear ganglion	Neuron/ganglial cell
41	**12.2**	Cochlear spiral ganglion neuron 3	Cochlear spiral ganglion neuron 2 Mdga1 Tmem108			Spiral ganglion neuron | Cochlear ganglion	Neuron/ganglial cell
6	**10.1**	Cochlear sulcus cell 1	Cochlear inner/outer sulcus cell Prss36 Hmcn1	Root	Root	Border cell/Claudius cell | Spiral sulcus	
7	**10.2**	Cochlear sulcus cell 2	Cochlear inner/outer sulcus cell Adamtsl1 Col11a1	Root	Root	Border cell/Claudius cell | Spiral sulcus	
9	**10.3**	Cochlear sulcus cell 3	Cochlear inner/outer sulcus cell Rasgef1b Lmo3	Root	Root	Border cell/Claudius cell | SPIRAL sulcus	
5	**21**	Cochlear supporting cell	Cochlear supporting cell Gpc5 Otogl		Root/Deiter	Supporting cell of cochlea | Epithelium of cochlear duct	
16	**13.2**	Unknown 1	Unknown Dnah12 Rgs22			?? |??	
17	**13.6**	Unknown 2	Unknown Bpifa1			?? | Cochlear modiolus	Mixed

### Cluster markers

NS-Forest was used to identify the minimum number of necessary and sufficient markers for each cluster (Aevermann et al., 2021). NS-Forest was performed with the number of trees set to 1,000 and the number of genes to test set to 6.

### Statistical analysis

**Figure 2:**
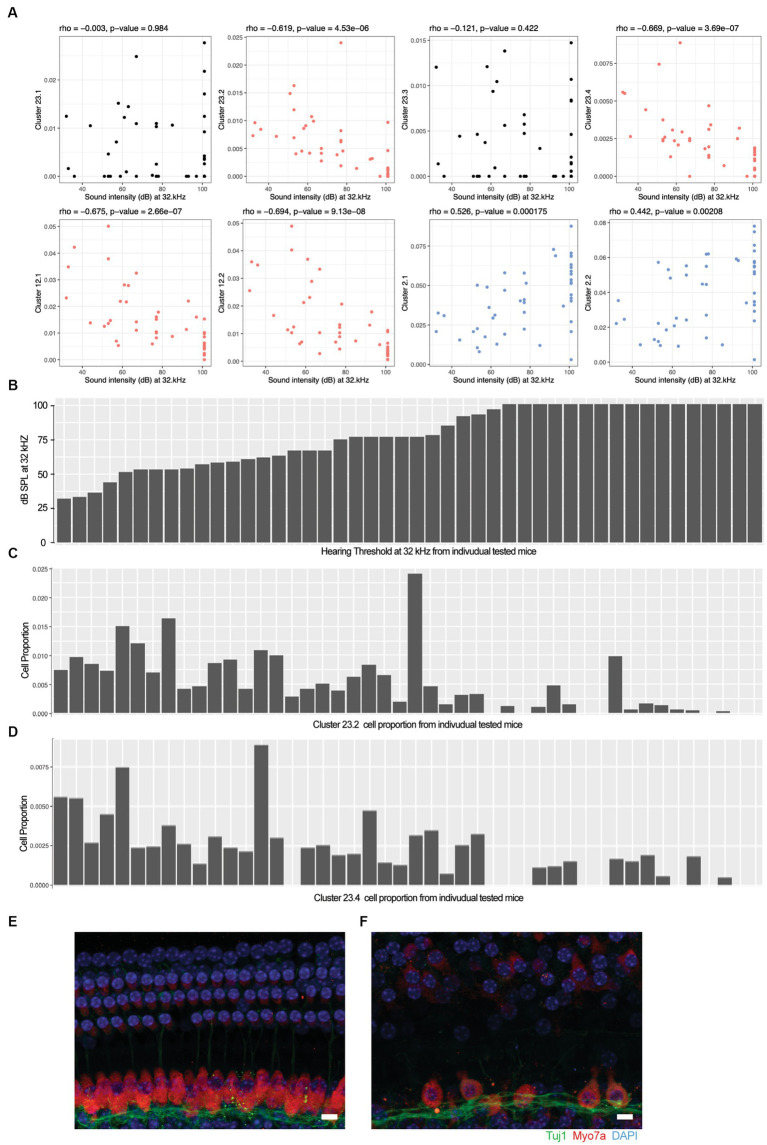
Loss of specific cochlear cell types with age-related hearing loss. The proportion of each cell type in the 48 outbred mouse cochlea was compared against the ABR sound intensity score at different sound frequencies. **(A)** Correlation between selected cell type proportions (*y*-axis) and ABR sound intensity thresholds at 32 kHz (*x*-axis). Hair cell Clusters 23.2 and 23.4 and ganglion neuron Clusters 12.1 and 12.2 show significant inverse correlations with intensity threshold (red). Basal cell Clusters 2.1 and 2.2 show significant positive correlation with intensity threshold (blue). In contrast to the inverse correlation observed with Clusters 23.2 and 23.4, hair cell Clusters 23.1 and 23.3 show no correlation with intensity threshold. Spearman’s rho correlation and value of *p* are show at the top of each plot. **(B)** Sound intensity threshold of individual mice ranked in order of hearing loss. **(C)** Proportion of Cluster 23.2 cells in individual mice. **(D)** Proportion of Cluster 23.4 cells in individual mice. Whole mount projections of organ of Corti corresponding to areas encoding 16 kHz frequency show that mice with all frequencies profound hearing loss **(F)** exhibit inner and outer hair cells loss that worsen through higher frequencies while the organ of Corti from mice with normal hearing **(E)** show intact structure and no apparent cells loss. Scale bar 10 um.

We calculated the Spearman’s rho correlation between the hearing acuity ABR score (*x*-axis in Figure 2A) and the cell population proportion (*y*-axis in Figure 2A) in each mouse. The correlation rho is reported at the top of each panel, capturing the association between the hearing acuity and cell proportion. A hypothesis test of rho = 0 (i.e., no correlation) vs. rho ≠ 0 for statistically significant correlation is applied and the *p* value is reported at the top of each panel. A significant positive correlation rho > 0 is indicated by blue, and a significant negative correlation rho < 0 is indicated by red.

### Multiplexed error-robust fluorescence *in situ* hybridization on cochlear tissue

Multiplexed error robust fluorescence *in situ* hybridization samples were prepared in accordance with company instructions (Vizgen, Cambridge, MA, United States). Briefly, C57Bl/6 J mice cochleae of postnatal day 5 and 2 months were harvested and fixed with 4% paraformaldehyde (PFA) in 0.1 M phosphate-buffered saline (PBS; pH 7.4) at 4°C overnight. Samples were dehydrated in graded-sucrose series (10 and 20% for 30 min, and 30% for overnight at 4°C) with RNase inhibitor [New England Biolabs (NEB), M0314L, Ipswich, MA, United States]. Samples were placed in a cryomold and embedded with O.C.T. compound (Sakura Finetek, Torrance, CA, United States), then frozen with dry ice/ethanol bath. The embedded tissue was sectioned into 10 μm thick slices using a Leica CM1860 (Leica Biosystems, Nussloch, Germany) cryostat, and 3–5 cochlear mid-modiolar sections were mounted onto a center of MERSCOPE slide glass (Vizgen, #20400001, United States). The mounted sections were washed with 0.1 M PBS, then permeabilized in 70% ethanol at 4°C for 24 h. The cell boundary staining was performed by using a primary antibody mix (Vizgen, #20300010, United States), followed by a secondary antibody mix (Vizgen, #20300011, United States) for 1 h at 23°C, respectively. Stained samples were incubated with an encoding probe [MERSCOPE 140 Gene Panel Mix (Vizgen, #20300006, United States)] for 36 h at humidified 37°C cell culture incubator. After post-encoding hybridization wash with formamide wash buffer (Vizgen, #20300002, United States), samples were embedded with a gel embedding solution [gel embedding premix (Vizgen, #20300004, United States), 5 mL; 10% ammonium persulfate solution (Millipore-Sigma, 09913-100G, Burlington, MA, United States), 25 μL; N,N,N′,N′-tetramethylethylenediamine (Millipore-Sigma, T7024-25ML, United States), 2.5 μL]. For tissue clearing, samples were incubated in digestion premix (Vizgen, #20300005, United States) with RNase inhibitor (NEB, United States) for 1 h at 23°C, followed by clearing premix [clearing premix (Vizgen, #20300003, United States), 5 mL; proteinase K (NEB, P8107S, United States), 50 μL] for 48 h at humidified 37°C cell culture incubator. After the tissue became transparent, samples were washed with the wash buffer (Vizgen, #20300001, United States) and incubated with 4′,6-diamidino-2-phenylindole (DAPI) and polythymine (polyT) staining reagent (Vizgen, #20300021, United States) for 15 min with agitation. Images were taken by using MERSCOPE (Vizgen, United States). DAPI/polyT and cell-boundary staining 2 was utilized for the cell segmentation parameter respectively, then image processing analysis was done on the MERSCOPE. The images were visualized and analyzed on the MERSCOPE Visualizer (Vizgen, United States).

## Results

Carworth Farms White mice are a genetically diverse outbred mouse population distinguished by degrading linkage disequilibrium (LD) between nearby alleles and shorter LD ranges compared to other commercially available inbred mouse strains (Rice and O’Brien, 1980; Chia et al., 2005), which makes them ideal models to capture high-resolution mapping in genome-wide association studies (GWAS). Our ongoing efforts to phenotype the hearing function in CFW mice have categorized them into eight distinct patterns of hearing, ranging from normal hearing, to moderate mid- or high-frequency hearing loss, to profound hearing loss at all frequencies, with each of those hearing patterns worsening with age (Du et al., 2022).

**Figure 3:**
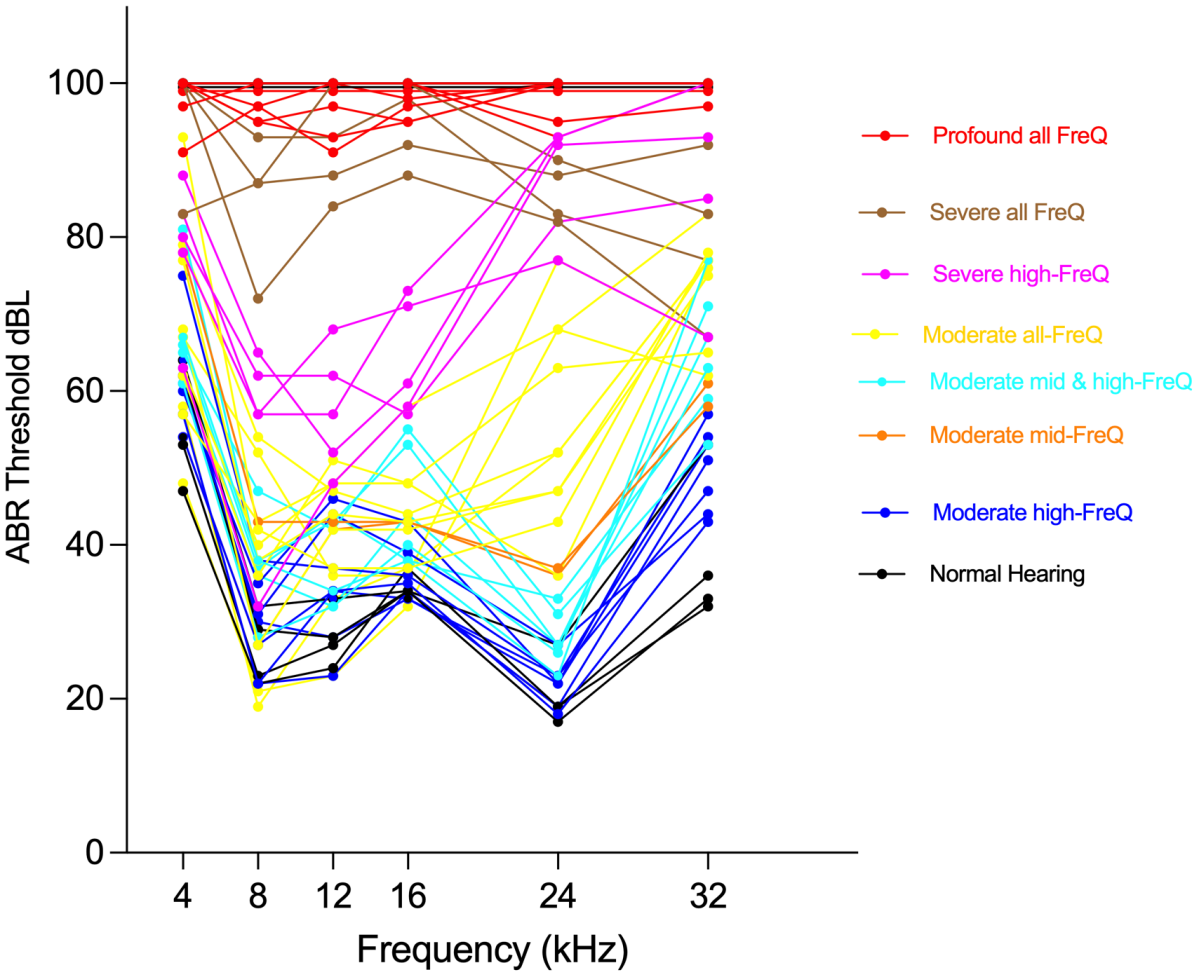
Phenotyping CFW outbred mice shows eight distinct hearing patterns. Individual Auditory Brain Response (ABR) thresholds plots for mice selected to represent CFW cohort hearing patterns for cochlear tissue collection and snRNA-seq analysis.

A group of 48 mice were selected to represent those hearing patterns (Figure 3), based on their proportion in the general CFW cohort, to explore the molecular and cellular correlates of hearing loss using single nucleus RNA sequencing (snRNA-seq) of dissected cochlea. During the dissection of inner ear tissue, special care was given to quickly process the samples while avoiding any shear force. The vestibular parts of the inner ear, including utricle, saccule, and semicircular canal ampulla, were removed before collecting cochlear tissue. snRNA-seq processing was chosen over scRNA-seq to avoid the stress responses induced during the single cell dissociation procedure and to provide for a more unbiased representation of cell types from solid tissues (Bakken et al., 2018).

Iterative unsupervised clustering was used to group snRNA-seq transcriptional profiles into transcriptome clusters. The initial Leiden community detection yielded 26 clusters, a subset of which (13) were processed through a second round of sub-clustering based on Silhouette score and manual inspection to yield a final collection of 60 transcriptome clusters (Figure 1A). The similarity relationships between the clusters were determined using hierarchical clustering and the relative abundance of each putative cell type determined (Figure 1B). The hierarchical clustering results confirmed the transcriptional relationships between the initial clusters and the sub-clusters with common origins from the second round of clustering.

As an initial approach toward determining which cell types correspond to these transcriptomic clusters, we examined the expression levels of several genes known to be expressed in specific ear cell types (Figure 1B). The organ of Corti supporting cell gene Epyc (Hanada et al., 2017) is uniquely expressed in the two related Clusters 10.1 and 10.2. The inner ear progenitor gene Lgr5 (Chen et al., 2021) is uniquely expressed in Cluster 6. The spindle-root gene Slc26a4 is uniquely expressed in the two related Clusters 19.1 and 19.2 (Nishio et al., 2016; Jean et al., 2023). The Kcnq1 marginal cell gene and the Slc26a7 Reissner’s membrane gene are preferentially expressed in Cluster 5 and Cluster 4, respectively. The hematopoietic stem cell-related gene Runx1 (de Bruijn and Dzierzak, 2017) is expressed in 12 related clusters. The intermediate cell genes Kcnj10 (Marcus et al., 2002) and Met (Shibata et al., 2016) are uniquely and preferentially expressed in Cluster 9. The glial and Schwann’s cell genes Mpz, Mbp, and Plp1 are expressed in the related Clusters 25, 17.1, 17.2, and 18. The hair cell (HC) genes Xirp2 (Scheffer et al., 2015a) and Cabp2 (Yang et al., 2016) are expressed in related Clusters 23.1, 23.2, 23.3, and 23.4. The spiral ganglion neuron (SGN) genes Slc4a4 (Grandi et al., 2020) and Grm8 (Sun et al., 2021) are expressed in related Clusters 12.1 and 12.2. The basal cell gene Cldn11 (Gow et al., 2004) is expressed in related Clusters 2.1 and 2.2. Finally, the supporting fibrocyte genes Slco1c1 (Ng et al., 2021) and Cald1 (Scheffer et al., 2015b) are expressed in 17 related clusters. While the expression pattern of known marker genes was useful in providing an initial annotation and validation of the snRNA-seq analysis, there were many examples where finer grained cell type distinctions were identified from the unsupervised clustering results.

**Figure 4:**
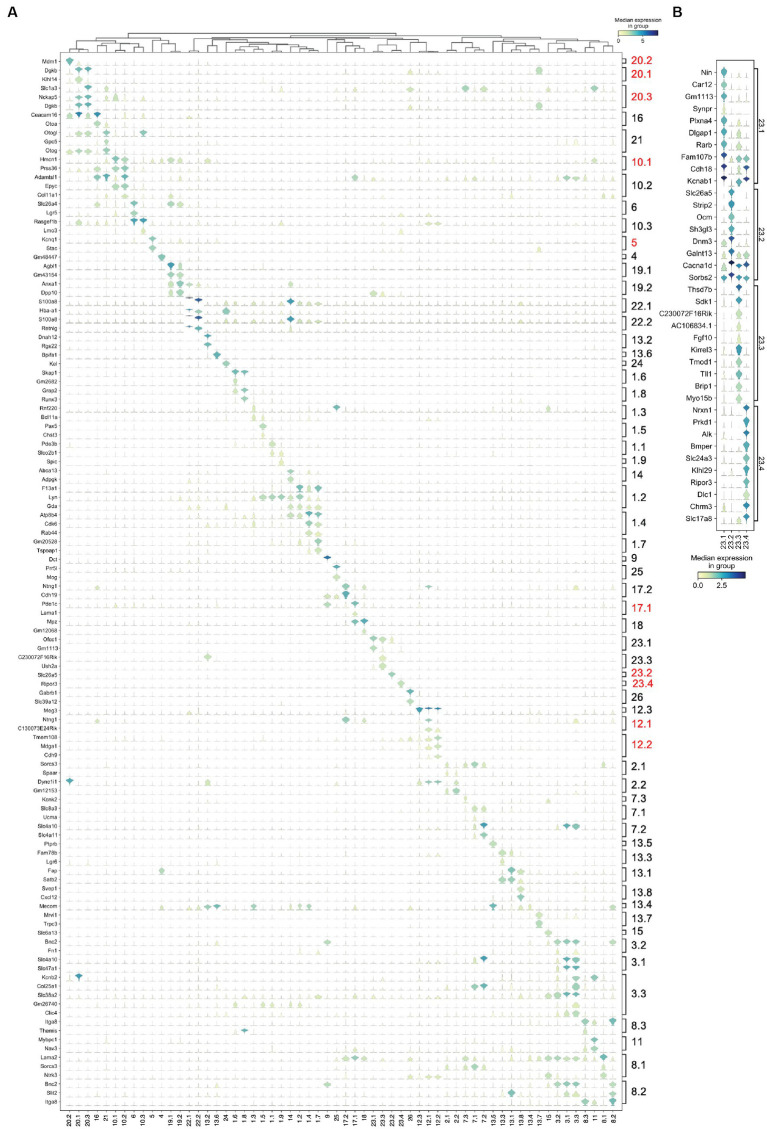
Cell type-specific marker genes. NS-Forest was used to identify the minimum set of necessary and specific marker genes for each cell type, with gene expression (rows) across each transcriptomic cluster (columns) illustrated in violin plots. **(A)** Minimum NS-Forest marker genes identified from the complete cell-by-gene expression matrix, with diagonal expression patterns and little off-diagonal expression showing marker gene specificity. In addition to the minimum site of marker genes, NS-Forest also produces an extended set of 10 genes selected based on binary expression (on–off) patterns in the target cluster. **(B)** Top 10 binary genes were identified within the four Cluster 23 iterative subclusters using the pooled Cluster 23 subcluster cell-by-gene expression matrix as input, showing strong and distinct binary expression patterns, especially for Clusters 23.3 and 23.4.

Therefore, to further extend this cell type classification, the NS-Forest algorithm was used to identify the minimum sets of marker genes for each cluster. NS-Forest uses random forest machine learning and a binary expression scoring method to identify necessary and sufficient marker genes, optimally capturing the essence of cell type identity (Aevermann et al., 2018, 2021). NS-Forest analysis of the 60 cell type clusters yielded 117 marker genes with high cell type specificity as illustrated by the diagonal expression pattern of markers across the clustered dataset (Figure 4A) and the relatively high F-beta values of classification accuracy (Supplementary Table 1). For example, HC Clusters 23.2 and 23.4 showed F-beta values of 0.93 and 0.88 using the single cell type markers Slc26A5 (Yamashita et al., 2015) and Ripor3, respectively. For the other hair cell clusters, combinatorial expression of two marker genes gave optimal classification accuracy of Fbeta = 0.85 with Gm1113 and Ofcc1 markers for Clusters 23.1 and markers Ush2a and C230072F16Rik for Cluster 23.3. NS-Forest marker genes for the SGN populations included Ntng1, Mdga1, Cdh9, and Meg3, which have been shown to be among the highest differentially expressed genes during the diversification process of SGN (Petitpré et al., 2022).

NS-Forest also produces an extended set of marker genes that show specific binary expression patterns across the cell clusters. In the case of the four HC clusters (Clusters 23.1, 23.3, 23.2 and 23.4), the genes Slc26a5, Ocm, and Cacnald encoding for Prestin (Yamashita et al., 2015), Oncomodulin (Sakaguchi et al., 1998), and Calcium voltage dependent channel (Cav1.3; Chen et al., 2012) respectively, are highly expressed in Cluster 23.2 suggesting this group is an outer hair cell (OHC) population (Figure 4B). Cluster 23.4 shows specific expression of Slc17a8 (Ruel et al., 2008) and Nrxn1 (Mozhui et al., 2011), known inner hair cell markers (IHC) encoding the Vesicular Glutamate Transporter-3 and a presynaptic adhesion protein, respectively. Cluster 23.3 shows specific expression of Myo15b, while Cluster 23.1 shows high expression of Kcnab1, which encodes a potassium voltage-gated channel, a hair cells marker conserved across species (Janesick et al., 2021) and shown to be more IHC specific in mature mouse cochlea (Liu et al., 2014; Bi et al., 2022). Genes recently validated as HC gene markers in newborn cochlea (Kolla et al., 2020) were not identified in our dataset, suggesting that those markers may be more specific for early postnatal stages.

To extend these granular cell type annotations further, the transcriptomics clusters were compared against five recently released snRNA and scRNA postnatal dataset (Supplementary Table 2) hosted by the gEAR database^2^ using FR-Match, a cluster-to-cluster cell type matching algorithm that incorporates shared information among cells to determine the relationship between two transcriptome clusters (Zhang et al., 2021) using the cluster-specific marker genes identified by NS-Forest to provide a reduced dimensional feature space and support their statistical comparison. A match was considered high confidence if a match was found in both directions (query to reference and reference to query) and/or if a match was found to the same cell type reported in two or more gEAR datasets; a match was considered low confidence if there was a match in only one dataset in only one direction. The FR-Match comparisons show that 29 clusters were matched to gEAR cell types with high confidence (Supplementary Figure 1; Table 1). The analysis results confirmed the classification of strial cells (marginal, intermediate, and basal) and spindle-root populations with high confidence (up to eight matches). The clusters corresponding to cochlear outer hair cells, glial/Schwann cells, immune cells, and a subset of fibrocyte populations were also confirmed with high confidence, albeit with lower numbers of matches. The cell populations characterized as Hensen’s cells, Dieters cells, and pillar cells and the remaining fibrocytes populations were matched with lower confidence. The results of FR-Match analysis were also consistent with the cluster hierarchy, highlighting the advantage of using the FR-Match to identify corresponding cell types.

**Figure 5:**
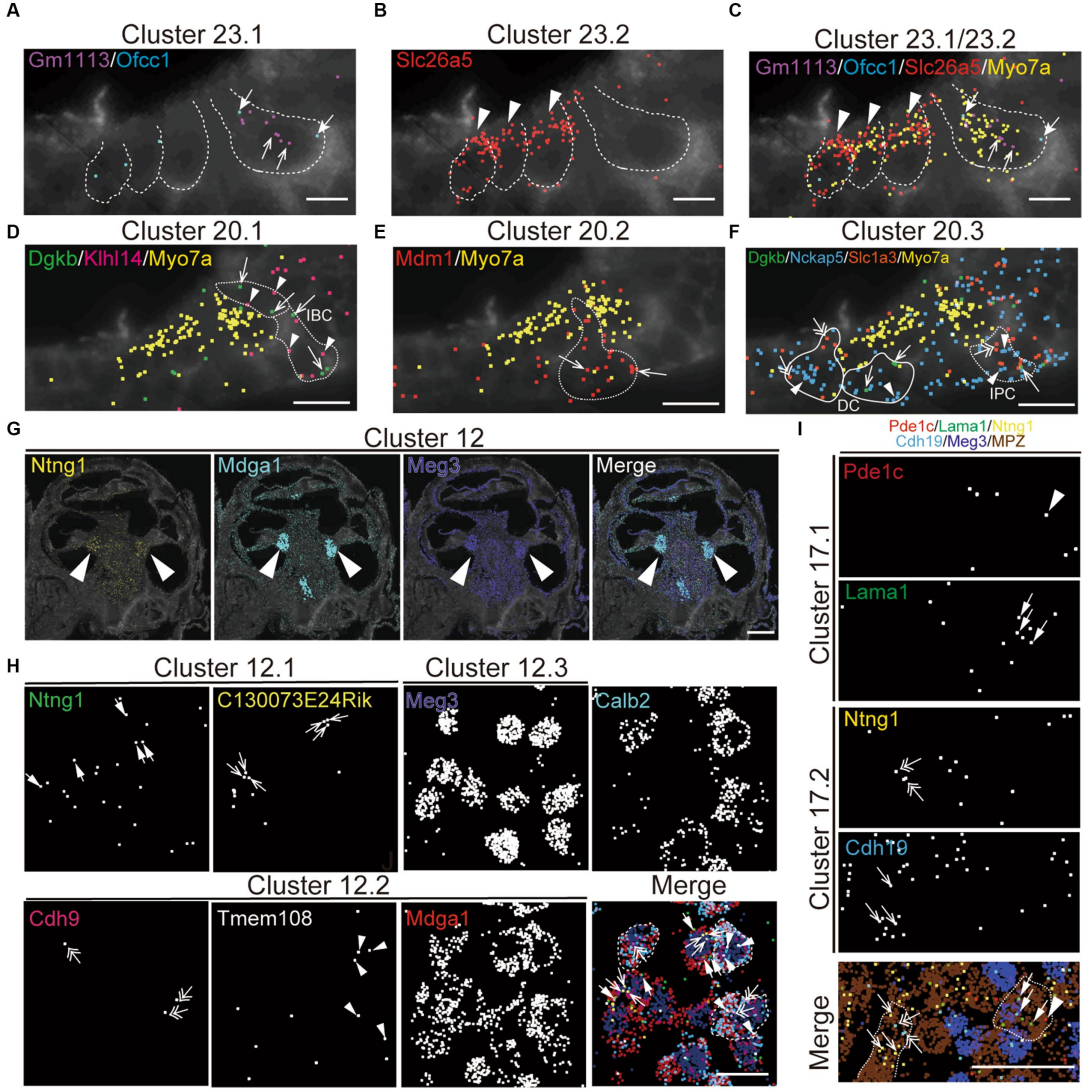
Spatial organization of cell types in the mouse cochlea. MERFISH images with selected marker genes for Cluster 23 (cluster of hair cell; **A–C**), Cluster 20 (cluster of supporting cell; **D–F**), Cluster 12 (cluster of spiral ganglion cell; **G,H**), and Cluster 17 (cluster of Schwann cell; **I**) on the cochlear mid-modiolar cryosections at P4. Each pseudo-color dot represent transcripts of different marker genes and is merged with cell-boundary staining in dark gray. Cell boundaries are depicted by white or white-dashed line. Myo7a was used for pan-hair cell marker gene (yellow; **C–F**). **(A)** Cluster 23.1 for inner hair cell (IHC) with Gm113 (purple, arrow), Ofcc1 (light blue, thick arrow). **(B)** Cluster 23.2 for outer hair cell (OHC) with Slc26a4 (red, arrowhead). **(C)** Cluster 23.4 for unknown hair-cell subtype with Ripor3 (white, arrow). **(D)** Cluster 20.1 for inner border cell (IBC) with Dgkb (green, arrow) and Klhl14 (magenta, arrowhead). **(E)** Cluster 20.2 for pillar cell with Mdm1 (red, arrow). **(F)** Cluster 20.3 for inner phalangeal cell (IPC), Deiter’s cell (DC) and IBC with Dgkb (green, arrow), Nckap5 (blue, arrowhead), and Slc1a3 (orange, double arrow). **(G)** Localization of marker genes for cluster 12 in cochlear spiral ganglion cells (arrow) with Ntng1 (yellow), Mdga1 (light blue), and Meg3 (dark blue). **(H)** High magnification view of cluster 12.1 with Ntng1 (green, thick arrow), C130073E24Rik (yellow, arrow), of cluster 12.2 with Cdh9 (magenta, double-head arrow), Tmem108 (white, arrowhead), and Mdga1 (red), and of cluster 12.3 with Meg3 (dark blue) and merged image with pseudo colors. Calbindin2 (calb2) is a known marker gene for type1a spiral ganglion neuron. Representative cell boundaries for Cluster 12.2 was depicted by white-dashed line. **(M)** Images for cluster 17.1 with Pde1c (red, arrowhead) and Lama1 (green, thick arrow), and for cluster 17.2 with Ntng1 (yellow, double-head arrow) and Cdh19 (light blue, arrow). Meg3 (dark blue) and Mpz (brown) indicate spiral ganglion cells and Schwann cells, respectively. Sale bars; 15 μm **(A–F)**, 250 μm **(G)**, and 25 μm **(H–J)**.

To validate the specificity of NS-Forest cell-type-specific marker expression and to characterize the spatial relationships between the cell types, the recently developed multiplexed error-robust fluorescent *in situ* hybridization technique (MERFISH), a single-molecule imaging approach that allows identification and spatial localizations of RNA transcripts for several different cell types (Moffitt and Zhuang, 2016; Zhuang, 2021), was used in cross-sections from P5 cochleae. The NSForest cell-type specific marker genes (Figure 4A) were utilized as MERFISH probes to confirm the consistency between the cell-type annotations and transcript localizations. For example, marker genes for hair cell clusters localized to inner hair cells (IHC; Cluster 23.1, Gm1113+/Ofcc1+; Figures 5A,C), outer hair cells (OHC; Cluster 23.2, Slc26A5+; Figures 5B,C). Moreover, the MERFISH imaging confirmed the annotation of some subcluster categories with the lower confidence FR-Match results, such as border cells for Cluster 20.1 (Dgkb+/Klhl14+; Figure 5D), inner and outer pillar cells for Cluster 20.2 (Mdm1+; Figure 5E), Deiter’s cell and inner phalangeal cell for Cluster 20.3 (Dgkb+/Nckap5+/Slc1a3+; Figure 5F), where the localization pattern and clustering matched conventional anatomical localization nomenclature in the cochlea. Interestingly, some marker genes localized to the same anatomical cell types but indicated different cell populations among the subcategories, such as Cluster 12 for spiral ganglion cells (Figures 5G,H) and Cluster 17 for Schwann cells (Figure 5I). Cluster 12.2 cells strongly expressed Calbindin2 (Calb2) known for a marker of type1a spiral ganglion neuron (Sun et al., 2018). These data suggested that the NS-Forest algorithm successfully identified unique marker genes for the transcriptomic clusters and the spatial transcriptomic analysis verified the FR-match annotations based on anatomical and physiological properties of cochlear cells.

For further evaluation of the proportion of cell types present, we investigated the spatial organization of the scala media containing the organ of Corti, which is the receptor organ for hearing, by MERFISH (Supplementary Figures 2A,B). P5 aged mice were used to due to the difficulties in obtaining sufficient material in adult mice and to generate an atlas of cell types unbiased by age. In addition to hair cell and supporting cell clusters (Figure 5), we successfully identified various cell clusters in the cochlear duct, such as epithelial cells of Reisner membrane (Cluster 4, Soc26a7+/Tmem72+; Supplementary Figure 2C), marginal cells (Cluster 5, Kcnq1+/Stac+), and intermediate cells (Cluster 9, Dct+) in the stria vascularis (Supplementary Figure 2D), spindle cell (Cluster 19.2, Anxa1+/Dpp10+; Supplementary Figure 2E), root cells in the spiral prominence (Cluster 6, Lgr5+/Slc26a4+; Supplementary Figure 2F), inner and outer sulcus cells (Cluster 10, Epyc+/Gata3+; Supplementary Figure 2G), fibrocyte in the tympanic covering layer of basilar membrane (Cluster 11, Mybpc1+/Nav3; Supplementary Figure 2H), and interdental cells in the spiral limbus (Cluster 16, Otoa+; Supplementary Figure 2I). Notably, the clustering successfully distinguished transcriptional profiles even among rare cell types in the spiral prominence of the cochlea, including spindle cells (Cluster 19) and root cells (Cluster 6). Moreover, these clusters in the neonatal mouse cochlea were matched and confirmed with the adult mouse cochlea (Supplementary Figure 3). These data demonstrate that the snRNA-seq approach robustly profiled cell type-specific transcriptomes in the cochlear tissue.

By combining the information derived from manual marker gene evaluation, gEAR reference matching, and MERFISH localization, cell type names and definitions for the 60 transcriptomics clusters were determined (Table 1). In order to provide for more descriptive cell types names for each of the transcriptomic clusters, we adopted a convention established by the ontology development community for defining and naming cell types derived from single cell transcriptomics experiments for incorporation into the Provisional Cell Ontology (Bakken et al., 2018; Tan et al., 2021), incorporating information about anatomic location, parent cell class, and specific marker gene combinations to provide for unique cell type names and experimentally useful definitions (Table 1; Supplementary Table 1) to serve as future references.

To evaluate the cellular correlates of the loss of hearing acuity in this outbred population, the auditory brainstem response (ABR) thresholds were correlated with the cell type proportions in the 48 outbred CFW mice with three patterns observed (Figure 2A). For example, the proportion of cells in Clusters 23.2, 23.4, 12.1, and 12.2 showed an inverse correlation with ABR threshold (Figure 2A, red; Figures 2B–D), whereas the proportion of cells in Clusters 2.1 and 2.2 showed a positive correlation (Figure 2A, blue), while most clusters showed no correlation (Figure 2A, black and Supplementary Figure 3). In support of this observation, whole-mount immunohistochemistry showed that OHC and IHC structures are normal in mice with good hearing (Figure 2E) compared to aged mice with poor hearing (Figure 2F), demonstrating that hair cell loss is a primary driver of hearing loss in CFW mice (Wu et al., 2020).

In comparing the cell type proportions with each other (Supplementary Figure 4), all cell types that show proportional loss with hearing loss are positively correlated with each other [e.g., Cluster 23.2 with Clusters 23.4, 12.1, and 12.2 (top row, left)], with the correlation strongest for cell types that are closely related to each other (e.g., Cluster 12.1 and 12.2, third row); all cell types that show proportional increases with hearing loss are positively correlated with each other [e.g., Cluster 2.1 with Clusters 2.2, 19.1, and 19.2 (fifth row, middle)]; all of the cell types that show proportional increases with hearing loss are negatively correlated with cell types that show proportional loss with hearing [e.g., Cluster 2.1 with Clusters 23.2, 23.4, 12.1, and 12.2 (fifth row, left)]; cell types that show no correlation with hearing loss show no correlation with cell types that show some correlation with hearing loss [e.g., Cluster 23.3 with Clusters 23.2, 23.4, 12.1, 12.2, 2.1, 2.2, 19.1, and 19.2 (bottom row, left and middle)]. Interestingly, although the proportions of hair cell Clusters 23.1 and 23.3 do not correlate with hair cell clusters 23.2 and 23.4, they show strong correlation with each other. These results suggest that hearing loss is associated with specific concerted changes in cell populations in this outbred population and that two specific types of HC populations and two SGN populations are still preserved despite hearing loss.

## Discussion

In this manuscript we have described the first snRNA-seq study of the aged adult outbred mouse cochlea correlated with auditory physiological data. In contrast to a recent study, using scRNAseq, which identified 27 cell types within the cochlea of a single mouse strain, C57BL/6J (Sun et al., 2023), we report the identification of 60 distinct cell types in this outbred mouse model using snRNA-seq to avoid the stress responses induced during the single cell processing procedure and to provide for a more unbiased representation of cell types from solid tissues (Bakken et al., 2018).

Presbycusis, or ARHL, is the most common sensory disorder in man and is characterized by reduced hearing sensitivities and speech understanding, particularly in noisy environments. Classically described, there are four subtypes: sensory, neural, stria and related areas and mixed expression, each associated with a corresponding cellular senescence (Schuknecht and Gacek, 1993). The findings reported here confirm the complex nature of ARHL regarding the survival/senescence of specific cell types. It is well known that SGN numbers decrease with age and are responsible for mixed and sensory forms of hearing loss including their ultimate decline after synaptic ribbon loss (Roux et al., 2006; Fernandez et al., 2015). In this study, two of the four SGN clusters decreased with increasing hearing loss, and yet, two subtypes of SGN cells identified in this analysis did not. It is also well known that HC loss is a common finding in most acquired hearing loss, yet, of the four subtypes of HCs detected in our in-depth analysis, only two declined with hearing loss. We also observed that pillar and inner border cell populations declined with hearing loss resulting in a loss of cytoarchitecture in the organ of Corti. Elevated hearing thresholds were associated with loss of marginal cell clusters in the stria vascularis, the portion of the ear responsible for maintenance of the endocochlear potential. The increased number of basal cells and spindle cells positively correlating with higher hearing thresholds suggests a compensatory role for these cells in maintaining the endocochlear potential with aging (Hibino and Kurachi, 2006; Szeto et al., 2022). The increased numbers of macrophages positively correlated with hearing loss likely represents an inflammatory response to the age-related cochlear stressors (Rai et al., 2020; Hough et al., 2022; Noble et al., 2022) which correlate with findings from Liu et al. on affected cellular pathway in aged mice (Liu et al., 2022). Finally, our analysis identified seven separate clusters of fibrocytes with two out of the seven declining in numbers with hearing loss.

The use of snRNA-seq to identify and quantify cell types in the cochlea of elderly mice with varying levels of age-related hearing loss (ARHL) identified 10 cell types whose proportions inversely correlated with hearing acuity, including two hair cell types (cochlear outer hair cell Slc26a5 and cochlear hair cell Ripor3), two spiral ganglion neuron types (cochlear spiral ganglion neuron C130073E24Rik Ntng1 and cochlear spiral ganglion neuron Mdga1 Tmem108), one Schwan cell (cochlear Schwan cell Pde1c Lama1), four border cell types (cochlear inner/outer sulcus Prss36 Hmcn1, cochlear border cell Dgkb Klhl14, cochlear pillar cell Mdm1, and cochlear phalageal cell Dgkb Nckap5), and one marginal cell type (cochlear marginal Stac Kcnq1; Supplementary Table 3). Each of these distinct cell types is characterized by the expression of specific combinations of marker genes. Since these marker genes show very specific expression in the cell type that they may play a critical role in the function of that cell type. For example, the two marker genes for cochlear marginal Stac Kcnq1 cells are both involved in ion channel function that regulate plasma membrane potential (Schroeder et al., 2000; Wong King Yuen et al., 2017) and are likely to be essential for the proper functioning of the cell. Since these cell types are critical for maintaining hearing with age, it is possible that their marker genes would also be essential for hearing. Indeed three of the 19 marker genes for these ARHL-associated cell types show genetic associations with hearing deficits (Supplementary Table 3). SGN type I and type have also been recently analyzed for Prph (Elliott et al., 2021) and Calb2 (Siebald et al., 2023). Kvlqt1 (aka Kcnq1) −/− mice are completely deaf due to defects in inner ear development (Lee et al., 2000). Mutations in KVLQT1 in humans cause Jervell and Lange-Nielsen (JLN) syndrome, an inherited autosomal recessive disease characterized by a congenital bilateral deafness associated with QT prolongation and ventricular arrhythmias (Neyroud et al., 1997). Mutations in PDE1C are associated with an autosomal dominant form of nonsyndromic postlingual progressive deafness in human (Wang et al., 2018). Mutations in SLC26A5 (aka Prestin) are associated with familial nonsyndromic hearing loss in humans (Liu et al., 2003). Homozygous Slc26a5 mutant mice showed a loss of outer hair cell electromotility *in vitro* and a loss of cochlear sensitivity *in vivo* without disruption of mechanoelectrical transduction in outer hair cells (Liberman et al., 2002). The finding that some of these cell type specific marker genes show genetic association with hearing deficits supports the observation that loss of these specific cell types would lead to ARHL.

Interestingly, marker genes specific for other cell types that did not correlate with ARHL in our mouse model also show genetic associations with certain types of hearing deficits (Supplementary Table 4). For example, although the proportion of cochlear hair cell Ush2a C230072F16Rik did not diminish with ARHL, mutations in its marker gene USH2A causes Usher syndrome type IIa, an autosomal recessive disorder characterized by moderate to severe sensorineural hearing loss and retinitis pigmentosa (Eudy et al., 1998).

In conclusion, by combining information from manual marker gene annotation, gEAR reference data matching and MERFISH spatial transcriptomics analysis, 60 unique transcriptomic clusters were identified and annotated with cell type identities and specific marker gene characterization. Several of these specific cell types showed preferential loss in the aging cochlea that also correlated with quantitative measures of hearing loss. The genes specifically expressed in these cells could serve as candidate targets for novel therapeutics in the future.

## Data availability statement

The original contributions presented in the study are publicly available. This data can be found here: https://umgear.org//index.html?share_id=f526abfe&gene_symbol_exact_match=1.

## Ethics statement

The animal study was approved by UCSD Institutional Animal Care and Use Committee (IACUC) Protocol 17178. The study was conducted in accordance with the local legislation and institutional requirements.

## Author contributions

EB: Conceptualization, Data curation, Investigation, Methodology, Validation, Writing – original draft, Writing – review & editing. NT: Data curation, Formal analysis, Investigation, Methodology, Software, Visualization, Writing – original draft. MN: Conceptualization, Formal analysis, Investigation, Methodology, Validation, Writing – original draft. YN: Data curation, Formal Analysis, Validation, Writing – original draft. ED: Data curation, Validation, Writing – original draft. CD: Data curation, Validation, Writing – original draft. YZ: Data curation, Formal analysis, Investigation, Methodology, Software, Visualization, Writing – original draft. UM: Methodology, Project administration, Supervision, Writing – original draft. RS: Conceptualization, Funding acquisition, Investigation, Methodology, Project administration, Resources, Supervision, Writing – original draft, Writing – review & editing. RF: Conceptualization, Funding acquisition, Project administration, Resources, Supervision, Writing – original draft, Investigation, Methodology, Writing – review & editing.

## Footnotes

^2^ https://umgear.org
