# Supplementary material for: Cochlear transcriptome analysis of an outbred mouse population (CFW)
Source: Front Cell Neurosci. 2023 Nov 29;17:1256619. doi: 10.3389/fncel.2023.1256619 (PMC10716316; doi:10.3389/fncel.2023.1256619)
Supplement: Supplementary file 5 [file Data_Sheet_1.PDF]

Hierarchical cluster order	Cluster ID	Cluster name	Cell type name	FR-Match high confidence	FR-Match low confidence	MERFISH cell type | anatomic structure	enrichR
1	20.2	cochlear pillar cell	cochlear pillar cell Mdm1		root	inner / outer pillar cell | epithelium of cochlear duct	mixed
2	20.1	cochlear border cell	cochlear border cell Dgkb Klhl14		root	border cell of cochlea | epithelium of cochlear duct	mixed
3	20.3	cochlear phalageal cell	cochlear phalageal cell Dgkb Nckap5		root	inner phalangeal cell / Deiters' cell | epithelium of cochlear duct	
4	16	cochlear interdental cell	cochlear interdental cell Otoa Ceacam16		root/Deiter	interdental cell of cochlea | tympanic lip of limbus of osseous spiral lamina	lymphoid
5	21	cochlear supporting cell	cochlear supporting cell Gpc5 Otogl		root/Deiter	supporting cell of cochlea | epithelium of cochlear duct	
6	10.1	cochlear sulcus cell 1	cochlear inner / outer sulcus cell Prss36 Hmcn1	root	root	border cell / Claudius cell | spiral sulcus	
7	10.2	cochlear sulcus cell 2	cochlear inner / outer sulcus cell Adamtsl1 Col11a1	root	root	border cell / Claudius cell | spiral sulcus	
8	6	cochlear root cell	cochlear root cell Lgr5 Slc26a4	root	root	root cell | outer spiral sulcus	
9	10.3	cochlear sulcus cell 3	cochlear inner / outer sulcus cell Rasgef1b Lmo3	root	root	border cell / Claudius cell | spiral sulcus	
10	5	cochlear marginal	cochlear marginal Stac Kcnq1	marginal	marginal	strial marginal cell | stria vascularis of cochlear duct	epithelial
11	4	cochlear Reissner's membrane	cochlear Reissner's membrane Gm48447	Reissner's	Reissner's	epithelial cell | Membrane of Reissner	retinal/sensory
12	19.1	cochlear spindle 1	cochlear spindle Gm43154 Agbl1	spindle	spindle	spindle cell | spiral prominence of cochlear duct	
13	19.2	cochlear spindle 2	cochlear spindle Dpp10 Anxa1	spindle	spindle	spindle cell | spiral prominence of cochlear duct	
14	22.1	cochlear neutrophil 1	cochlear neutrophil S100a8 Hba-a1		neutrophil	neutrophil | cochlea	neutrophils
15	22.2	cochlear neutrophil 2	cochlear neutrophil S100a8 Retnlg	neutrophil	neutrophil	neutrophil | cochlea	
16	13.2	unknown 1	unknown Dnah12 Rgs22			?? | ??	
17	13.6	unknown 2	unknown Bpifa1			?? | cochlear modiolus	mixed
18	24	cochlear erythroblast	cochlear erythroblast Kel	erythroblast	erythroblast	erythroblast | cochlea	erythroblast
19	1.6	cochlear NK/T cell	cochlear NK/T cell Gm2682 Skap1			mature NK T cell | cochlear nerve	NK/T cell
20	1.8	cochlear CD8+ T cells	cochlear CD8+ T cells Runx3 Grap2			CD8-positive, alpha-beta T cell | cochlea	CD8+ T cells
21	1.3	cochlear leukocyte	cochlear leukocyte Rnf220 Bcl11a			leukocyte | cochlea	
22	1.5	cochlear B cell	cochlear B cell Chst3 Pax5	B cell	B cell	B cell | cochlear nerve	B cell
23	1.1	cochlear macrophage 1	cochlear macrophage 1	Macrophage	macrophage	tissue-resident macrophage | cochlea	various immune cell types
24	1.9	cochlear macrophage 2	cochlear macrophage 2 Spic			tissue-resident macrophage | cochlea	macrophage
25	14	cochlear neutrophil 3	cochlear neutrophil Abca13 Adpgk	neutrophil	neutrophil	neutrophil | cochlea	myeloid/neutrophil
26	1.2	cochlear neutrophil 4	cochlear neutrophil F13a1 Lyn			neutrophil | cochlea	

27	**1.4**	cochlear leukocyte	cochlear leukocyte Rab44 Cdk6			leukocyte | cochlea	
28	**1.7**	cochlear myeloid/neutrophil	cochlear myeloid/neutrophil Gm20528 Tspoap1			myeloid leukocyte | cochlea	myeloid/neutrophil
29	**9**	cochlear Intermediate	cochlear Intermediate Dct	Intermediate	intermediate	strial intermediate cell | stria vascularis of cochlear duct	melanocytes
30	**25**	cochlear oligodendrocyte	cochlear oligodendrocyte Prr5l Mog		oligodendrocyte	oligodendrocyte | cochlear nerve	oligodendrocyte
31	**17.2**	cochlear Schwann cell 1	cochlear Schwann cell Ntng1 Cdh19			Schwann cell | cochlear ganglion / cochlear nerve	stromal
32	**17.1**	cochlear Schwann cell 2	cochlear Schwann cell Pde1c Lama1			Schwann cell | cochlear ganglion / cochlear nerve	neuron
33	**18**	cochlear Schwann cell 3	cochlear Schwann cell Mpz Gm12068	glia	glia/Schwann cell	Schwann cell | cochlear ganglion / cochlear nerve	Schwan cell
34	**23.1**	cochlear inner hair cell	cochlear inner hair cell Gm1113 Ofcc1			cochlea inner hair cell | epithelium of cochlear duct	myocytes
35	**23.3**	cochlear hair cell 1	cochlear hair cell Ush2a C230072F16Rik			cochlea auditory hair cell | epithelium of cochlear duct	neuron/ganglion
36	**23.2**	cochlear outer hair cell	cochlear outer hair cell Slc26a5	outer hair cell	outer hair cell	cochlear outer hair cell | epithelium of cochlear duct	mixed
37	**23.4**	cochlear hair cell 2	cochlear hair cell Ripor3			cochlea auditory hair cell | epithelium of cochlear duct	mixed
38	**26**	cochlear astrocyte	cochlear astrocyte Gabrb1 Slc39a12		Hensens	astrocyte | cochlear nerve	astrocyte
39	**12.3**	cochlear spiral ganglion neuron 1	cochlear spiral ganglion neuron 3 Meg3			spiral ganglion neuron | cochlear ganglion	
40	**12.1**	cochlear spiral ganglion neuron 2	cochlear spiral ganglion neuron 1 C130073E24Rik Ntng1			spiral ganglion neuron | cochlear ganglion	neuron/ganglial cell
41	**12.2**	cochlear spiral ganglion neuron 3	cochlear spiral ganglion neuron 2 Mdga1 Tmem108			spiral ganglion neuron | cochlear ganglion	neuron/ganglial cell
42	**2.1**	cochlear basal 1	cochlear basal Sorcs3 Spaar	basal	basal	basal cell | spiral ligament	
43	**2.2**	cochlear basal 2	cochlear basal Dync1i1 Gm12153	basal	basal	basal cell | cochlear ganglion	
44	**7.3**	cochlear fibrocyte 1	cochlear fibrocyte Kcnk2	fibrocyte	fibrocyte	fibrocyte | spiral ligament	
45	**7.1**	cochlear fibrocyte 2	cochlear fibrocyte Slc8a3 Ucma	fibrocyte	fibrocyte	fibrocyte | spiral ligament	
46	**7.2**	cochlear fibrocyte 3	cochlear fibrocyte Slc4a10 Slc4a11	fibrocyte	fibrocyte	fibrocyte | spiral ligament	
47	**13.5**	cochlear endothelial	cochlear endothelial Ptprb	fibrocyte	fibrocyte	endothelial cell | spiral modiolar artery	ciliated epithelial/endothelial
48	**13.3**	cochlear ciliated epithelial 1	cochlear ciliated epithelial Lgr6 Fam78b	fibrocyte	fibrocyte	ciliated epithelial cell | ??	ciliated epithelial/osteocytes
49	**13.1**	cochlear ciliated epithelial 2	cochlear ciliated epithelial Satb2 Fap	fibrocyte	fibrocyte	ciliated epithelial cell | cochlear modiolus	ciliated epithelial
50	**13.8**	cochlear ciliated epithelial 3	cochlear ciliated epithelial Cxcl12 Svep1	fibrocyte	fibrocyte	ciliated epithelial cell | cochlear modiolus	ciliated epithelial/fibroblast
51	13.4	cochlear ciliated epithelial 4	cochlear ciliated epithelial Mecom	fibrocyte	fibrocyte	ciliated epithelial cell | ??	ciliated epithelial
52	**13.7**	cochlear smooth muscle cell	cochlear smooth muscle cell Mrvi1 Trpc3	fibrocyte	fibrocyte	smooth muscle cell | spiral modiolar artery	ciliated epithelial/smooth muscle
53	**15**	cochlear glial	cochlear glial Slc6a13	fibrocyte	fibrocyte	glial cell | cochlear nerve	glial
54	**3.2**	cochlear basal 3	cochlear basal Bnc2 Fn1	fibrocyte	basal	basal cell | cochlea	
55	**3.1**	cochlear basal 4	cochlear basal Slc47a1 Slc4a10	fibrocyte	basal	basal cell | cochlea	

<table>
<tr><td>56</td><td>3.3</td><td>cochlear basal 5</td><td>cochlear basal Clic4 Gm26740</td><td>fibrocyte</td><td>basal</td><td>basal cell | cochlea</td><td></td></tr>
<tr><td>57</td><td>8.3</td><td>cochlear fibrocyte 4</td><td>cochlear fibrocyte Themis Itga8</td><td>fibrocyte</td><td>fibrocyte</td><td>fibrocyte | cochlear ganglion</td><td></td></tr>
<tr><td>58</td><td>11</td><td>cochlear fibrocyte 5</td><td>cochlear fibrocyte Mybpc1 Nav3</td><td>fibrocyte</td><td>fibrocyte</td><td>fibrocyte | basilar membrane of cochlea</td><td>various stromal cell types</td></tr>
<tr><td>59</td><td>8.1</td><td>cochlear fibrocyte 6</td><td>cochlear fibrocyte Sorcs3 Lama2</td><td>fibrocyte</td><td>fibrocyte</td><td>fibrocyte | cochlear ganglion</td><td></td></tr>
<tr><td>60</td><td>8.2</td><td>cochlear fibrocyte 7</td><td>cochlear fibrocyte Itga8 Slit2</td><td>fibrocyte</td><td>fibrocyte</td><td>fibrocyte | spiral ligament / cochlear ganglion</td><td></td></tr>
</table>
