# Supplementary figures and images for: Cochlear transcriptome analysis of an outbred mouse population (CFW)
Source: Front Cell Neurosci. 2023 Nov 29;17:1256619. doi: 10.3389/fncel.2023.1256619 (PMC10716316; doi:10.3389/fncel.2023.1256619)

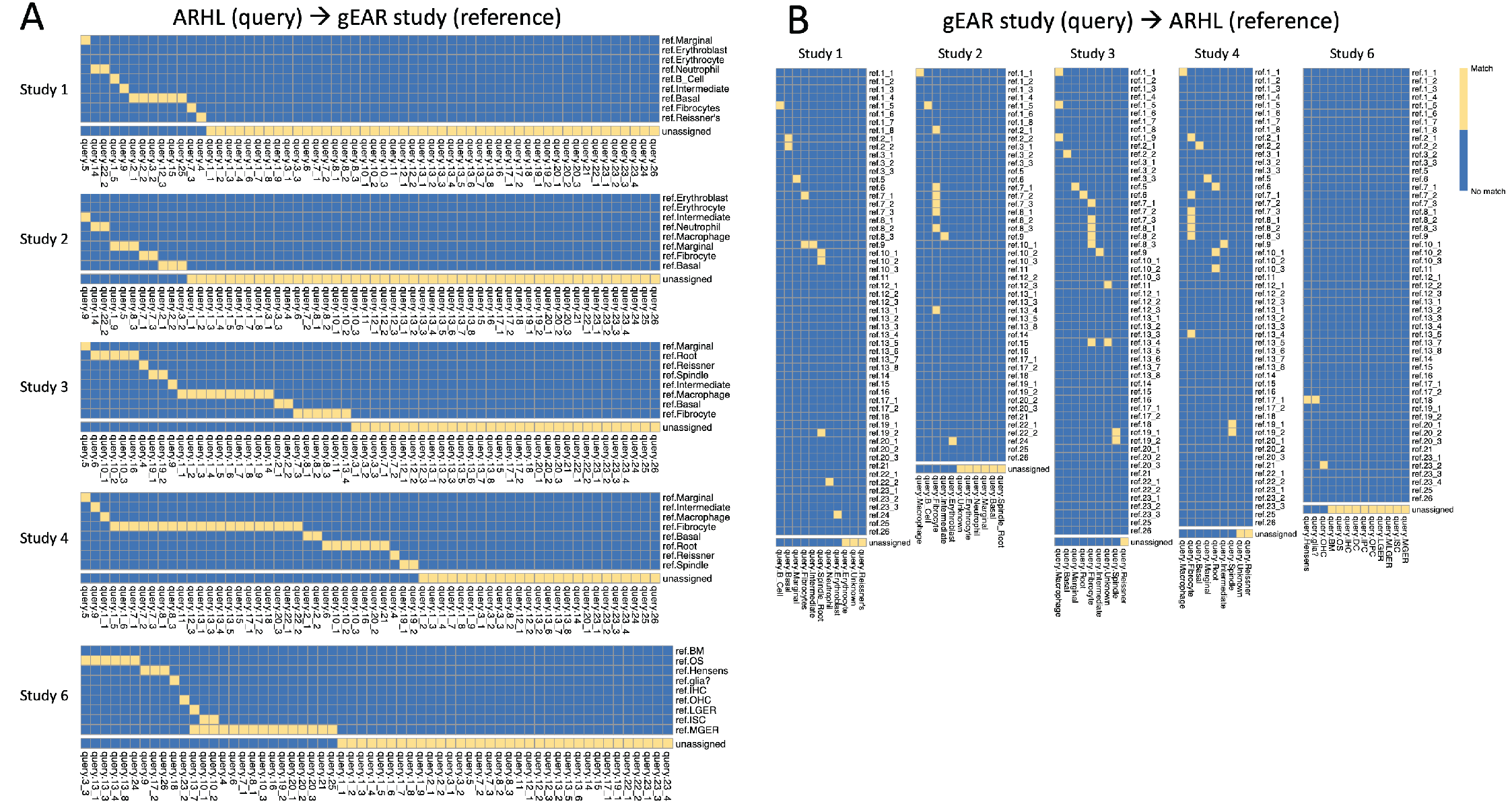

Supplement: Supplementary file 6 [file Image_1.TIF]

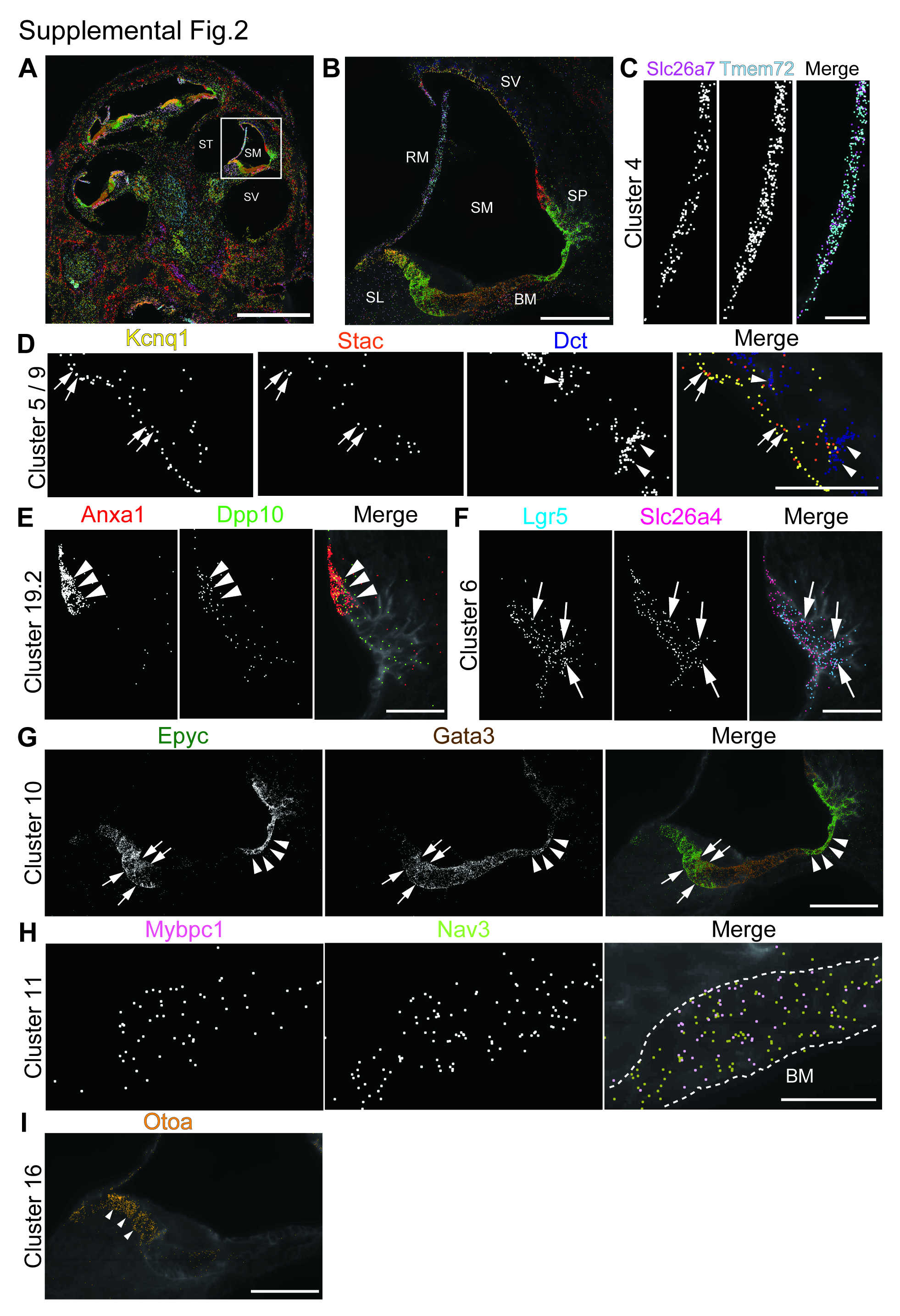

Supplement: Supplementary file 7 [file Image_2.TIF]

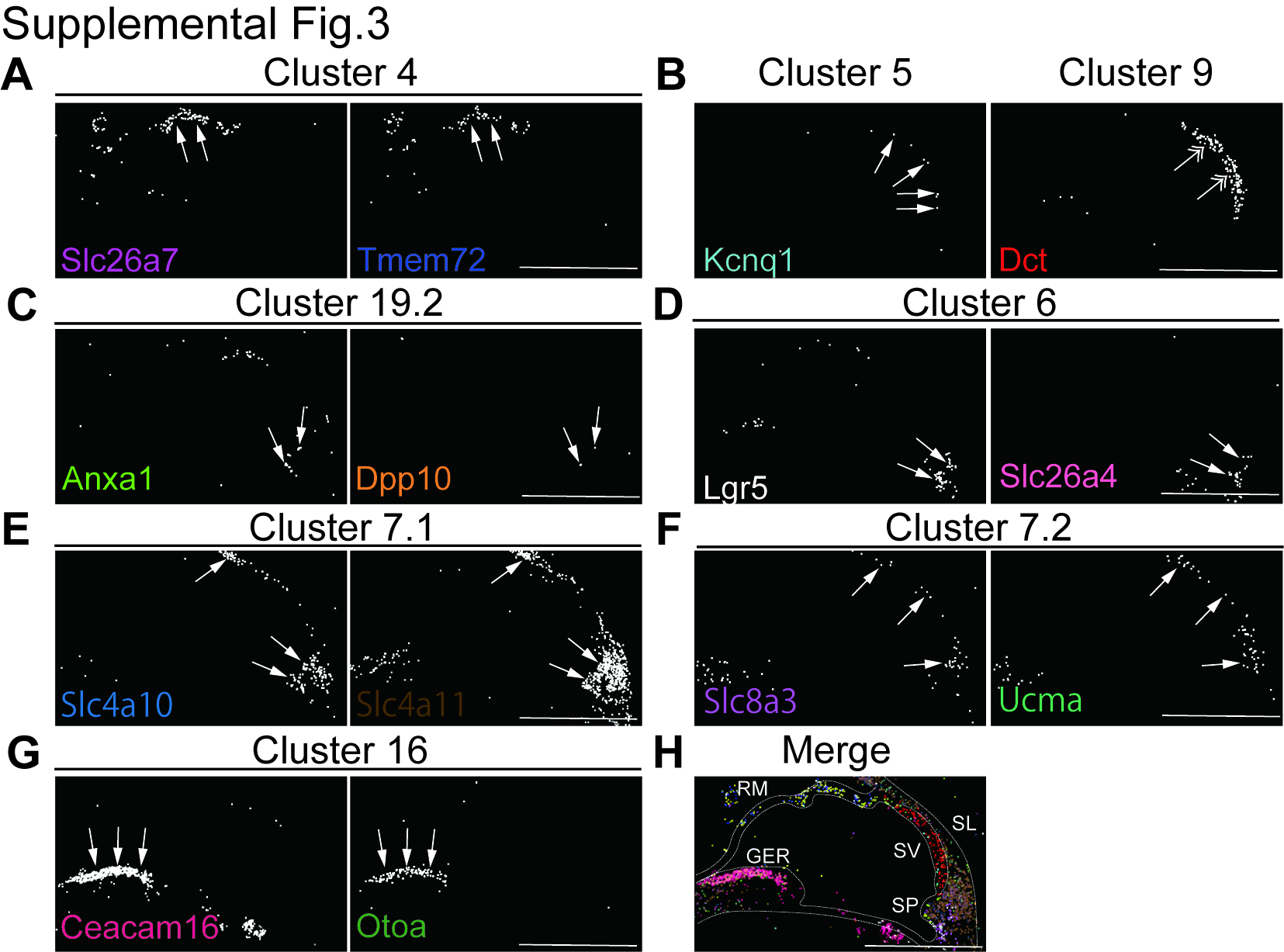

Supplement: Supplementary file 8 [file Image_3.TIF]

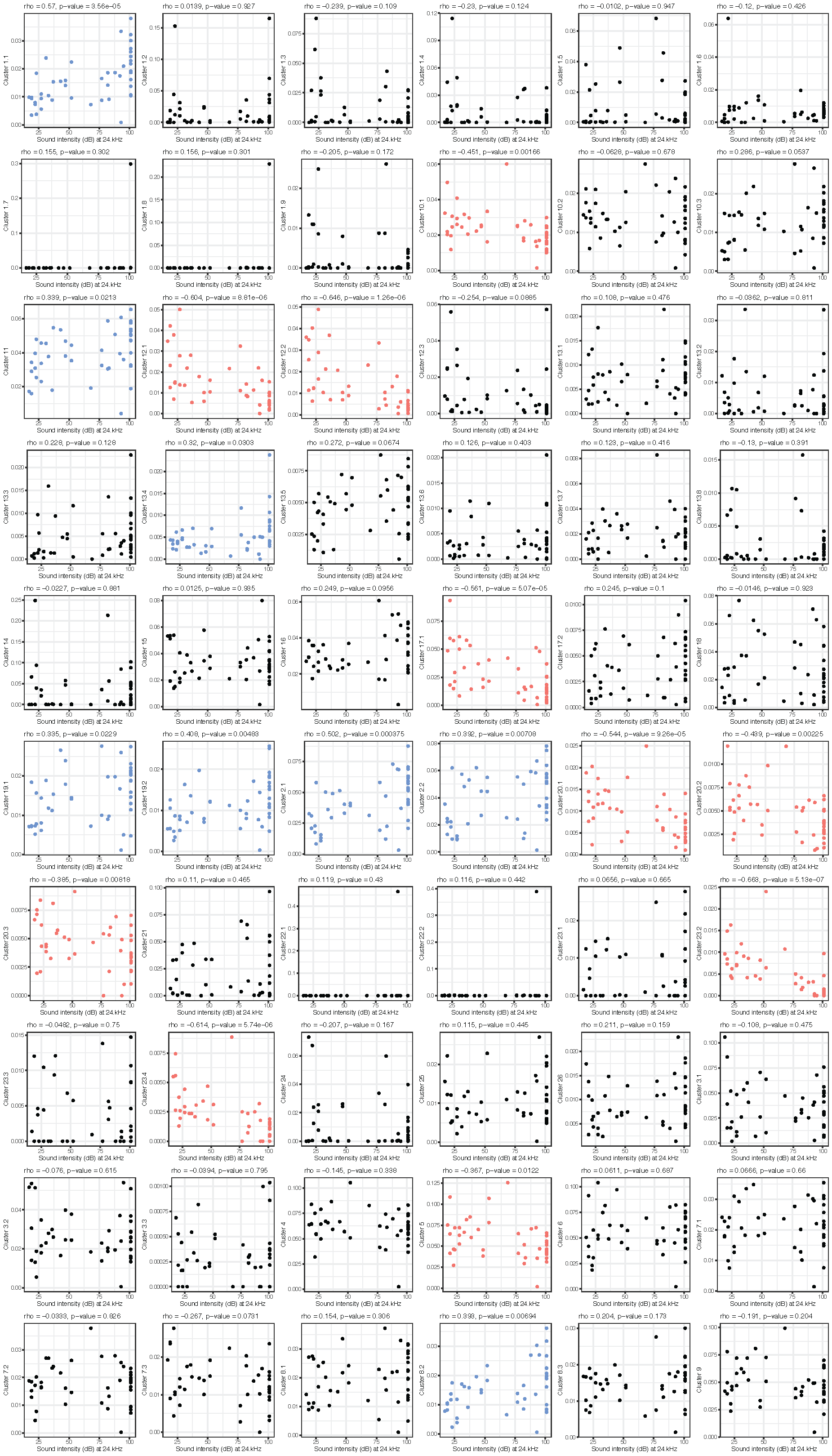

Supplement: Supplementary file 9 [file Image_4.TIF]

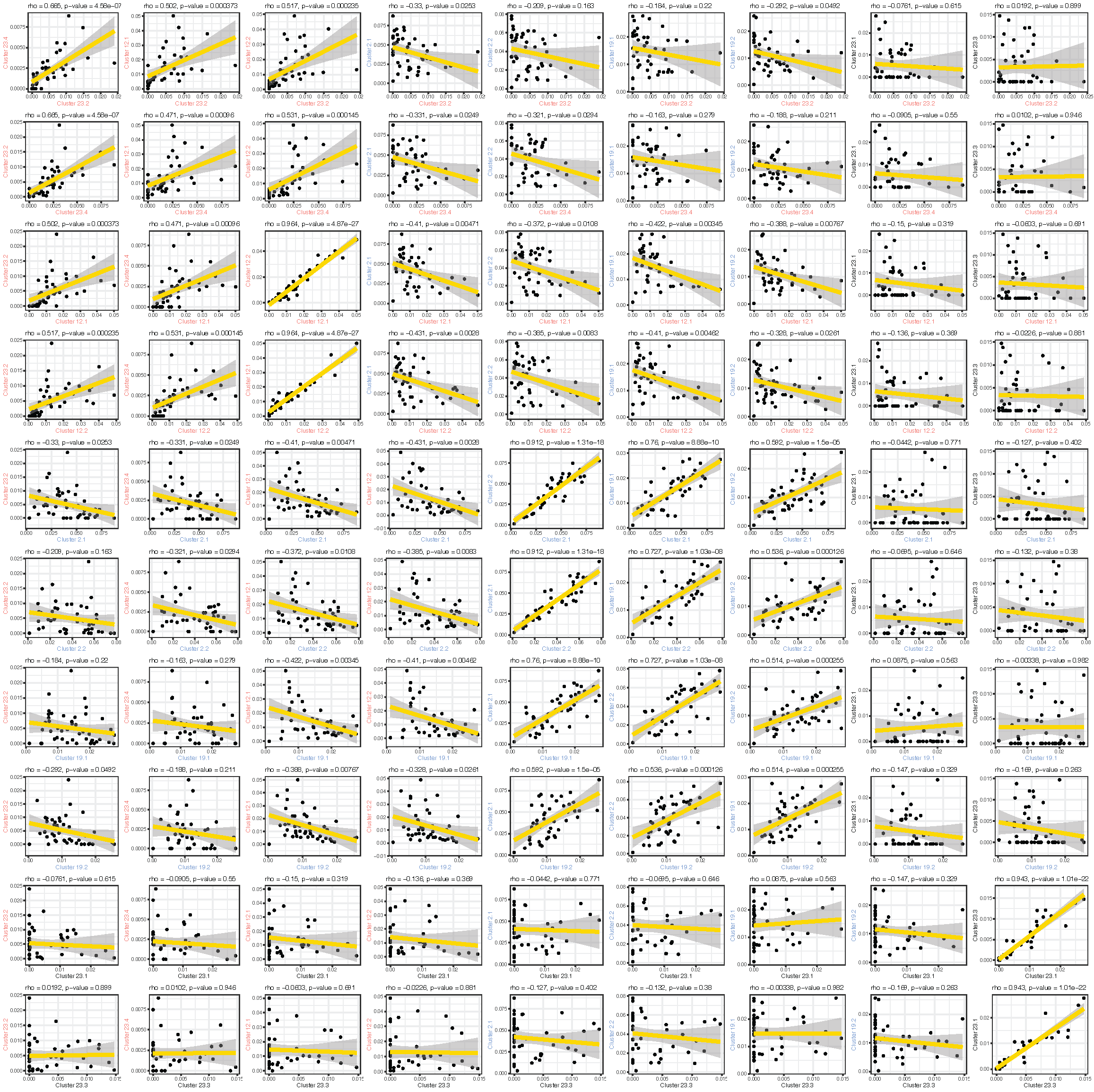

Supplement: Supplementary file 10 [file Image_5.TIF]

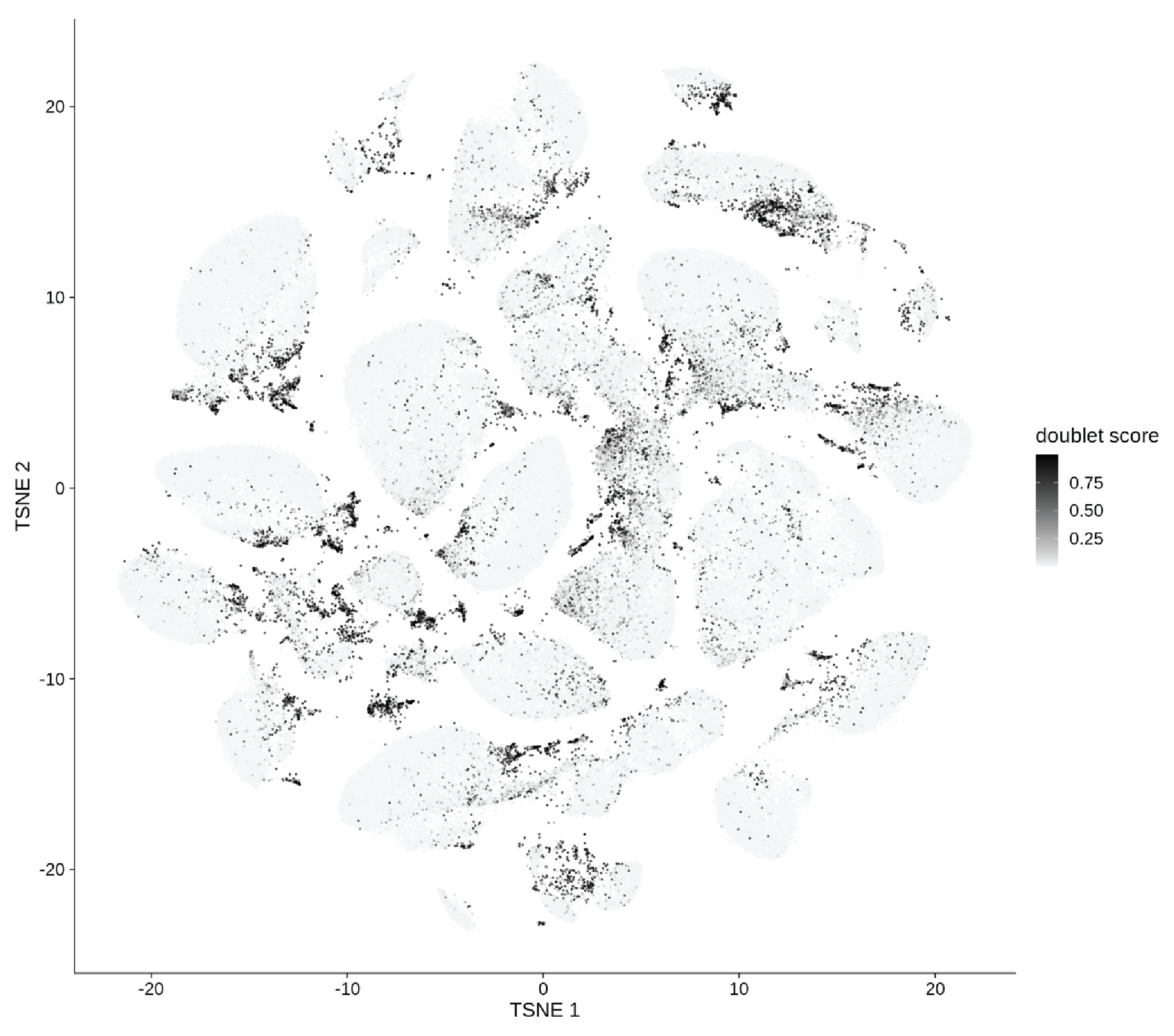

Supplement: Supplementary file 11 [file Image_6.TIF]

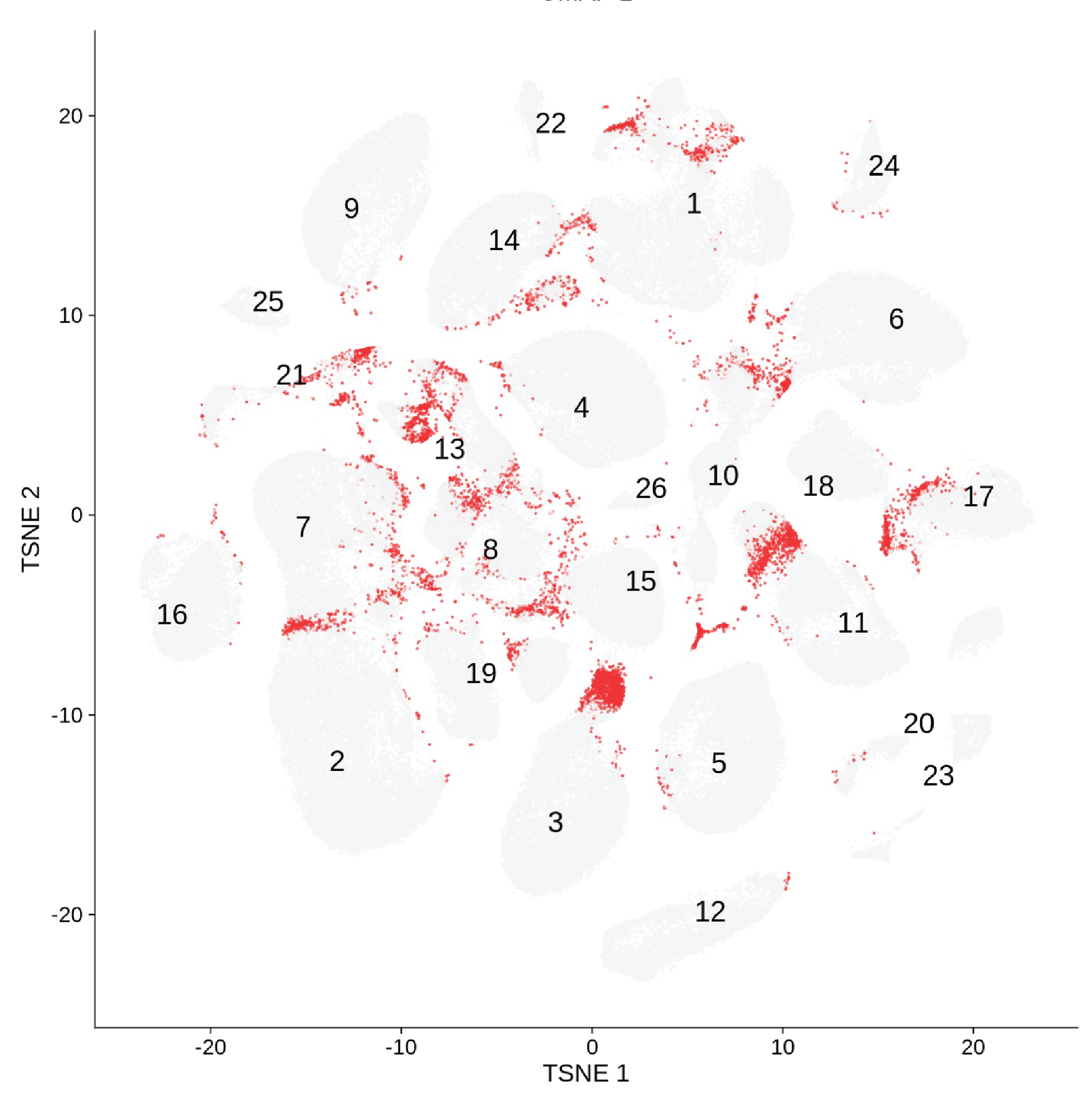

Supplement: Supplementary file 12 [file Image_7.TIF]

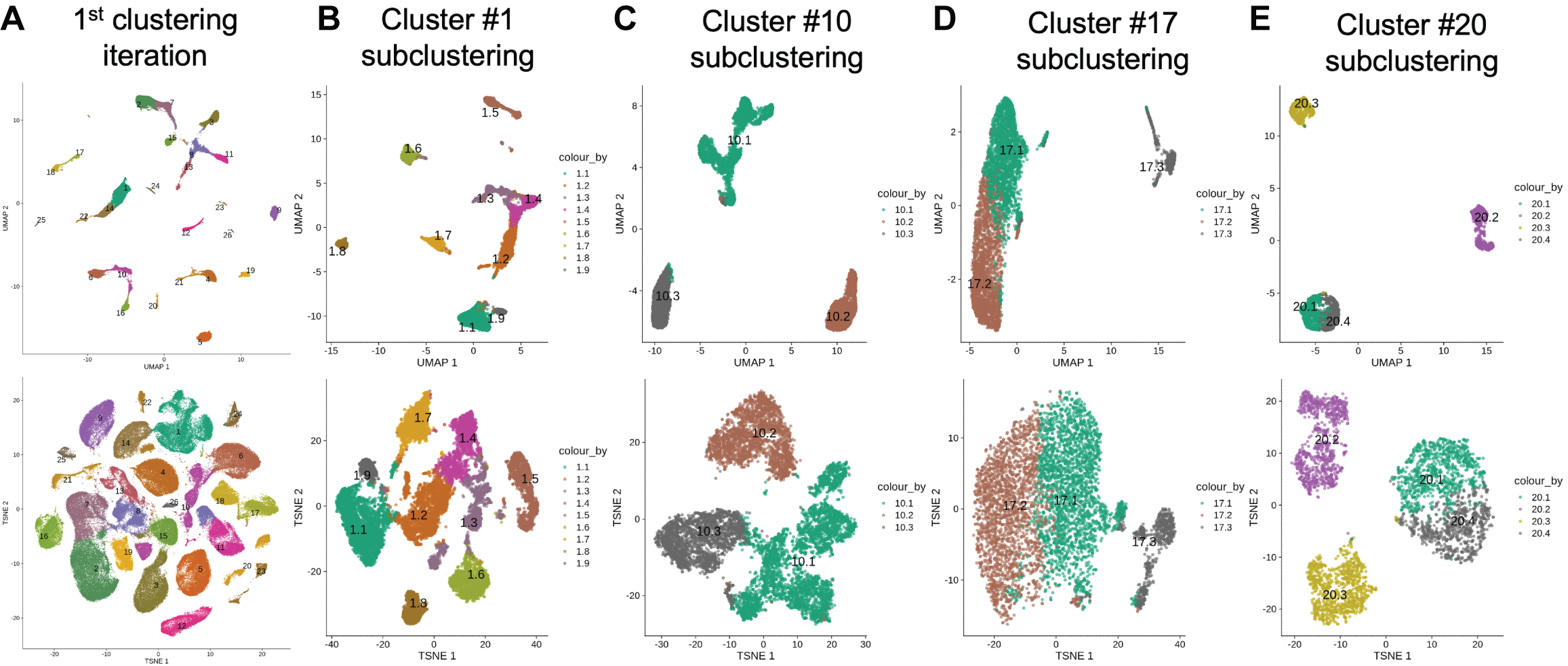

Supplement: Supplementary file 13 [file Image_8.TIF]
